# Cross-Neutralising Nanobodies Bind to a Conserved Pocket in the Hemagglutinin Stem Region Identified Using Yeast Display and Deep Mutational Scanning

**DOI:** 10.1371/journal.pone.0164296

**Published:** 2016-10-14

**Authors:** Tiziano Gaiotto, Simon E. Hufton

**Affiliations:** Biotherapeutics Group, National Institute for Biological Standards and Control, a centre of the Medicines and Healthcare Products Regulatory Agency, Blanche Lane, South Mimms, Potters Bar, Herts, EN6 3QG, United Kingdom; New York Blood Center, UNITED STATES

## Abstract

Cross-neutralising monoclonal antibodies against influenza hemagglutinin (HA) are of considerable interest as both therapeutics and diagnostic tools. We have recently described five different single domain antibodies (nanobodies) which share this cross-neutralising activity and suggest their small size, high stability, and cleft binding properties may present distinct advantages over equivalent conventional antibodies. We have used yeast display in combination with deep mutational scanning to give residue level resolution of positions in the antibody-HA interface which are crucial for binding. In addition, we have mapped positions within HA predicted to have minimal effect on antibody binding when mutated. Our cross-neutralising nanobodies were shown to bind to a highly conserved pocket in the HA2 domain of A(H1N1)pdm09 influenza virus overlapping with the fusion peptide suggesting their mechanism of action is through the inhibition of viral membrane fusion. We also note that the epitope overlaps with that of CR6261 and F10 which are human monoclonal antibodies in clinical development as immunotherapeutics. Although all five nanobodies mapped to the same highly conserved binding pocket we observed differences in the size of the epitope footprint which has implications in comparing the relative genetic barrier each nanobody presents to a rapidly evolving influenza virus. To further refine our epitope map, we have re-created naturally occurring mutations within this HA stem epitope and tested their effect on binding using yeast display. We have shown that a D46N mutation in the HA2 stem domain uniquely interferes with binding of R2b-E8. Further testing of this substitution in the context of full length purified HA from 1918 H1N1 pandemic (Spanish flu), 2009 H1N1 pandemic (swine flu) and highly pathogenic avian influenza H5N1 demonstrated binding which correlated with D46 whereas binding to seasonal H1N1 strains carrying N46 was absent. In addition, our deep sequence analysis predicted that binding to the emerging H1N1 strain (A/Christchurch/16/2010) carrying the HA2-E47K mutation would not affect binding was confirmed experimentally. This demonstrates yeast display, in combination with deep sequencing, may be able to predict antibody reactivity to emerging influenza strains so assisting in the preparation for future influenza pandemics.

## Introduction

Influenza A virus remains a persistent threat to public health resulting in 200,000–500,000 deaths worldwide every year [1]. Vaccination is the main treatment option however, the prediction of which viral strains will emerge and infect the human population, plus the timely generation of strain specific vaccines, remains a challenge. Neutralising antibodies against the main viral glycoprotein, hemagglutinin (HA), is the primary correlate of protection in humans [2]. Hemagglutinin can be classified into eighteen different subtypes in two antigenically distinct groups, group 1 (H1, H2, H5, H6, H8, H9, H11, H12, H13, H16, H17 and H18 subtypes) and group 2 (H3, H4, H7, H10, H14 and H15 subtypes), which in combination with nine different neuraminidase (NA) subtypes generate all known influenza viruses. The HA protein is a homotrimer of approximately 200kDa and is synthesised as a polypeptide HA0 that is post-translationally cleaved into two subunits, HA1 and HA2. The two domains are linked by a disulphide bond which fold into a structure comprising a highly variable globular head and a more conserved proximal stem domain [2, 3]. The pre-dominant host immune response is directed against the globular head [4] and this selective pressure drives the continuous antigenic changes of the influenza virus [5]. The high mutation rate and transmissibility means a new vaccine is required every year and make the discovery of new therapeutics which can overcome the ability of influenza to escape the human immune system an active area of research [6]. The structure of the membrane proximal stem region is significantly more conserved and this feature has led to the isolation of a number of cross-reactive monoclonal antibodies specific for group 1 [7–14], group 2 HAs [15–18] or both [19–21]. Two human monoclonal antibodies, CR6261 [22] and F10 [9], are perhaps the most notable examples and are being pursued as passive immunotherapeutics [23]. An interesting observation revealed by their crystal structures has shown that both these antibodies use only their heavy chain for recognition of a conserved epitope in the HA stem, with the light chain (LC) being superfluous to requirements. This suggests that ‘heavy chain only’ may be a preferred mode of binding to influenza HA as has been reported for HIV [24]. This has promoted interest in naturally occurring ‘heavy chain only’ antibodies such as those well documented in camelid species which are naturally devoid of a paired LC [8, 25, 26]. We have recently described five cross-neutralising single-domain antibodies (nanobodies) against 2009 pandemic H1N1 pandemic and highly pathogenic avian influenza (H5N1) virus. These nanobodies share the high selectivity, specificity and affinity of conventional antibodies however their small size, high stability, modular format and cleft binding properties are suggested to give distinct advantages over conventional antibodies as both immunotherapeutics and diagnostics [8].

Hemagglutinin specific monoclonal antibodies can inhibit infection by different mechanisms of action which include either blocking viral attachment to sialic acid residues of host cell surface proteins, interfering with the structural transition of HA that triggers membrane fusion activity in the endosomes, or by simultaneous inhibition of attachment and viral cell fusion [2]. The precise location of epitopes on HA targeted by cross-neutralising monoclonal antibodies is crucial in understanding their mechanism of action, their cross-reactivity and their relative susceptibility to loss of binding through antigenic changein a rapidly evolving virus.

The widespread use of *in vitro* display technologies has facilitated the isolation of large panels of recombinant antibodies against diverse targets including influenza [27]. These antibodies in turn require rapid, high-throughput and precise approaches to locate their epitopes. The ‘gold standard’ approach for mapping antibody epitopes is X-ray crystallography which defines interactions within the antibody-antigen complex to atomic resolution. However, the low-throughput, the need for highly pure reagents and the limited availability of expensive X-ray facilities are major limitations. In addition X-ray crystallography may not distinguish energetically important residues in the epitope from other residues which make up the broader antibody antigen interface. More general approaches use gene fragment libraries [28–30] or peptide arrays [31, 32] to locate antibody epitopes to linear stretches of protein sequence. Although these are potentially high-throughput they are generally confined to non-conformational epitopes and as over 90% of epitopes are discontinuous in the target amino acid sequence, their usefulness is clearly limited [33]. Mutational scanning is a more suitable approach to map conformational epitopes and involves the generation of a library of individual mutations with subsequent analysis for their specific impact on binding [34, 35]. However, this approach is still limited by the need to synthesize, express and purify each individual protein variant. This problem can be solved by using display technologies such as phage [36, 37], ribosome [38, 39] or yeast display [40, 41], where the whole library of mutations can be selected at once without the need to purify individual variants. Yeast display has emerged as a powerful technology for epitope mapping as its eukaryotic translation machinery acts as a quality-control for functionally folded protein variants. This means mutations which have more general structural effects on protein folding and stability do not get displayed and so focuses on the identification of mutations which directly impact antibody binding. In addition, simultaneous selection for both display and binding using flow cytometric cell sorting means each protein variant can be individually selected on the basis of multiple parameters [40–44]. Conventional sanger sequencing of protein variants with reduced binding, together with known structural information, allows the precise localisation of residues comprising conformational epitopes. Although this approach has proved successful, the small sample size possible with conventional DNA sequencing limits the assessment of the mutational landscape to key contact residues whereas mutations with more subtle effects on antibody binding may not be identified. The advent of deep sequencing has allowed a more comprehensive analysis of the whole repertoire of mutations [45, 46] displayed on the yeast cell surface in addition to identifying residues that are likely to have minimal effect on binding when mutated. This latter property has important implications when considering the scope of binding reactivity of individual monoclonal antibodies to a rapidly evolving target antigens.

Viral escape mutagenesis has been used to locate antibody epitopes on rapidly evolving viral antigens like influenza HA. However this is a very time consuming process requiring repeated cycles of growth in the presence of specific antibodies, sequencing of live virus to identify escape mutations followed by their re-introduction into infectious virus to confirm their significance. This approach has been successful in locating antibody epitopes on influenza HA but has been limited to those epitopes which can be mutated without interfering with viral infection such as those in the variable head domain [47–49]. Crucially, cross-neutralising antibodies which bind to epitopes which overlap functionally conserved regions, such as those in the HA stem domain, has proved challenging using this approach, as these residues are vital and cannot be easily disrupted without interfering with viral function [7, 9].

In this study we have used yeast display and deep mutational scanning to precisely map the epitopes of a panel of cross-neutralising nanobodies against A(H1N1)pdm09 and highly pathogenic avian influenza H5N1 [8]. Structure function aspects of these epitopes are discussed in terms of their mechanism of action, cross-reactivity profile and susceptibility to antigenic escape. We demonstrate how deep mutational scanning can define residues likely to have minimal effect on binding and present examples which highlight the potential of this approach to predict antibody binding to new viruses.

## Experimental Procedures

### Reagents and influenza antigens

Recombinant HA antigens from A/Solomon Islands/3/2006 (H1N1), A/South Carolina/1/1918 (H1N1), A/California/07/2009 (H1N1)pdm09, A/Brisbane/59/2007 (H1N1), A/New Caledonia/20/1999 (H1N1) and A/Vietnam/1194/2004 (H5N1) (eEnzyme) and A/Christchurch/16/2010 (H1N1) (Immunotech) were used. The virus antigen standard used in this study was derived from A/California/07/2009 (H1N1)pdm09 (National Institute for Biological Standards and Control, NIBSC). Expression and purification of single domain antibodies (sdAbs) R1a-G6, R1a-F5, R2b-D9, R2a-G8, R1a-B6, R1a-A5 and R2b-E8 was as described previously [8]. All sdAbs were also fused to a C-terminal c-Myc tag for detection. Commercial IgG antibodies FC41 (Ab00149-1.1) (Absolute Antibodies), RM10 (1055-RM10) (Sino Biologicals) and MIA-H7-334 (eEnzyme) were used as controls for correctly folded hemagglutinin (HA).

### Display of wild-type and chimeric hemagglutinin antigens on the yeast cell surface

A/California/07/2009 (H1N1)pdm09 hemagglutinin gene (Uniprot accession number C3W5X2) was codon optimized for yeast expression and synthesized (residues HA1 11–329 and HA2 1–184, H3 numbering [3]) (Integrated DNA Technologies). The construct was cloned as *Sfi*1/*Not*1 fragment into pTQ5 [50] to create plasmid pNIBS-5 which carries a SV5 tag instead of a c-Myc tag and transformed into *S*. *cerevisiae* EBY100 using a yeast transformation kit (Sigma). HAs from the viral strains (H5N1) A/VietNam/1203/2004 (Uniprot accession number Q6DQ34, residues HA1 11–329 and HA2 1–185, H3 numbering) and (H7N7) A/Netherlands/219/2003 (Uniprot accession number Q6VMK1, residues HA1 26–344 and HA2 1–189, H3 numbering) were similarly synthesised for expression in yeast. Stem/head chimeric HA genes were also designed and displayed (S1 Supporting Information). Standard procedure for growth and labelling yeast cells, media and buffer recipes for growth of yeast cultures were used [51] When stained with FC41, RM10, MIA-H7-334 and anti-SV5 commercial antibodies, the following reagents were used; goat anti-mouse DyLight633 (Thermo Scientific) for FC41 and MIA-H7-334, goat anti-rabbit AlexaFluor647 (Invitrogen) for RM10, and donkey anti-mouse IgG AlexaFluor488 (Invitrogen) for detection the SV5 tag. Staining with sdAbs carrying a c-Myc epitope tag was performed with polyclonal chicken anti-cMyc (Bethyl Laboratories) followed by donkey anti-chicken IgG AlexaFluor647 (Jackson ImmunoResearch). Stained cells were analysed on BDAria III flow cytometers (Becton Dickinson), and data analysed using BD Diva and FlowJo software.

### Assessing the sensitivity of sdAb binding to HA of heat and low pH

Yeast cells displaying HA from A(H1N1)pdm09 were incubated at 60°C for 30 minutes. The sample was then chilled on ice for 30 minutes and separately labelled with 100nM of R1a-F5, R1a-G6, R2b-E8, R2b-D9, R1a-A5, R1a-B6 and R2a-G8, or 1μg/ml of mouse anti-SV5 antibody. After washing, cells were stained with secondary reagents as previously described and mean fluorescence intensity (MFI) determined. The head-binding single domain antibody R1a-F5 was sub-cloned into the yeast display vector pNIBS-5 as a *Sfi*1/*Not*1 fragment and transformed into EBY100. The antigen standard A/California/07/2009 (H1N1)pdm09 at 100nM concentration in either 50mM Tris-HCl pH 8.0 or 100mM sodium acetate buffer pH4.8 was pre-incubated for 1 hour at room temperature followed by incubation with yeast cells displaying R1a-F5. After a further 30 minutes, cells were incubated at neutral pH containing sdAbs at 1μg/ml in PBS. sdAb binding was then assessed by labelling with anti c-Myc as described previously and analysis on a BD Canto II flow-cytometer.

### Construction and screening of random mutagenised HA library

A library of HA0 mutants was generated by error-prone PCR using oligonucleotides pTG6-linker-For (5’- tctgggggcggaggatctg -3’) and SV5-Rev (5’- agtccaaacccaacaatgggtttg -3’), ThermoPol polymerase (New England Biolabs) and an unequal molar concentration of deoxynucleotides; 0.5mM of dATP and dCTP, 0.1mM of dTTP and dGTP. 20 μg of error-prone PCR product was co-transformed with 20 μg of *Sfi*1/*Not*1 digested pNIBS-5 vector into EBY100 competent cells [52]. The final library size was determined through serial dilutions on selective plates. The yeast library was grown in selective medium for induction of HA display. 10^8^ cells were co-stained with 100nM sdAb, followed by anti-SV5/anti-cMyc antibodies and by fluorescent secondary reagents. Flow cytometric cell sorting was performed using BDAria III. A gate was chosen to sort cells for HA display (by virtue of anti SV5 signal) but absence of sdAb binding (lower right quadrant of a FACS dot plot). A second round was performed using the same sorting conditions, followed by a third round of positive sorting (upper right quadrant), labelling previous outputs with anti-SV5, and either 200nM of R1a-B6 for epitope mapping of head-binding sdAbs or 1μg/ml of RM10 for epitope mapping of stem-binding sdAbs, followed by staining with specific secondary reagents.

### Deep sequencing of selected yeast displayed library

The unselected yeast displayed library and the outputs following selection were processed and analysed through a MiSeq next generation sequencer (Illumina). Plasmid DNA was extracted from yeast cells using Zymoprep Yeast Plasmid Miniprep II kit (Zymoresearch), and a 294-bp fragment (HA1-Gly^303^– HA2-Asn^71^ H3 numbering) was amplified by PCR using two amplicon primers HA2_NGS_for (5’—tcgtcggcagcgtcagatgtgtataagagacagGGAAAATGTCCAAAATATGTAAAAAGC -3’) and HA2_NGS_rev (5’—gtctcgtgggctcggagatgtgtataagagacagTGGTTGAACTCTTTACCTACTGC—3’), including both gene-specific and adapter sequences (gene-specific sequence is shown in uppercase text). Amplification was performed using Phusion High-Fidelity DNA polymerase (New England Biolabs), and the products were purified and used as templates for barcoding PCRs using Nextera index kit (Illumina). Amplicons were purified using QIAquick PCR purification kit (Qiagen), quantified and quality-checked using a QuBit fluorometer (ThermoScientific) and a Agilent DNA 1000 Kit (Agilent Technologies), respectively. Samples were processed for deep sequencing reactions using a MiSeq Reagent Kit v2, 500-cycle (Illumina). The deep sequencing datasets are available through accession number PRJEB15301 at the European Nucleotide Archive website (http://www.ebi.ac.uk/ena/data/view/PRJEB15301).

### Sequence analysis and bioinformatics

Following de-multiplexing and trimming using Illumina sequencing software, pair-end reads were aligned using FLASH [53], and filtered by quality and length using web-based software Galaxy [54]. The generated files were then uploaded into Geneious software. After translation, the reads were grouped according to frequency and sequences either containing proline/cysteine substitutions or with more than one mutation were excluded from further analysis. Only sequences reaching an output frequency of greater than 0.5% were aligned to A(H1N1)pdm09 HA wild-type sequence to locate specific mutations. For each filtered read, we calculated the frequency (f) and the enrichment factor (E) relevant to the unselected library. We used reads from the output of the first round of selection to compile a list of mutations with an enrichment factor E≥5. Key residues of the antibody epitope were highlighted on A(H1N1)pdm09 HA crystal structure (PDB structure 3AL4) using Chimera software (https://www.cgl.ucsf.edu/chimera/). Yeast clones carrying single point mutations predicted to form the antibody epitope by deep sequencing were either identified by sequencing the entire HA gene of entire yeast clones or through synthesis using site-directed mutagenesis using a QuickChange II Site-Directed Mutagenesis Kit (Agilent) according to manufacturer’s instructions. Only mutants with single amino acid substitution in an otherwise wild-type HA sequence were chosen for experimental testing. Yeast clones were separately labelled for HA display and sdAb binding, and the extent of binding relative to HA display analysed by flow cytometry. The sdAb-binding mean fluorescence intensity (MFI) of each antibody-mutant HA pair was divided by the MFI value of the wild-type H1N1 HA, and the resulting ratio given as percentage values.

### Construction of rationally designed mutants

Datasets of full-length hemagglutinin sequences were downloaded from the Influenza Research Database (www.fludb.com) available in GenBank as of October 2014 using the following search criteria; protein data (virus type “A”, protein “HA”, host “human”), complete segments only and duplicate sequences removed. Dataset for H1N1, H5N1, H2N2 and H9N2 subtypes and were downloaded into Geneious software (http://www.geneious.com/) for global alignment within each HA specific subtype. Focusing on region Gly^1^-Asn^60^ (HA2 domain), we compared A(H1N1)pdm09 wild-type residues to aligned datasets. We identified positions with amino acid diversity and calculated the frequency of each naturally occurring amino acid. Only amino acids with a frequency greater than 3% are shown in S4 Table. Selected mutants were generated using QuickChange II Site-Directed Mutagenesis Kit (Agilent) following manufacturer’s instructions. HA mutants were checked by sequencing and plasmids transformed into EBY100 cells. The binding activity of sdAbs to HA mutants displayed on yeast was assessed as previously described.

### Analysis using surface plasmon resonance

For sdAb binding and affinity ranking against different recombinant HA a BIAcore T100 machine (GE Healthcare) was used in combination with a single cycle kinetics procedure [55]. In brief, purified recombinant HA from H1N1 strains between 1918 and 2010 (A/South Carolina/1/1918, A/New Caledonia/20/1999, A/Solomon Islands/3/2006, A/Brisbane/59/2007, A/California/07/2009, A/Christchurch/16/2010) and H5N1 subtype A/Vietnam/1194/2004 (Protein Sciences) were immobilised onto a BIAcore CM5 chip in 10mM sodium acetate pH 5.5 using an amine coupling kit (GE Healthcare). A concentration series of purified sdAbs was sequentially run over the different antigen surfaces ranging from 1nM to 10nM. A reference surface was subtracted prior to evaluation of the sensograms using the single cycle kinetics procedure of the BIAevaluation software (GE Healthcare) in combination with a 1:1 fitting model.

## Results

### Display of A(H1N1)pdm09 HA0 and characterization of single domain antibody binding

**Fig 1:**
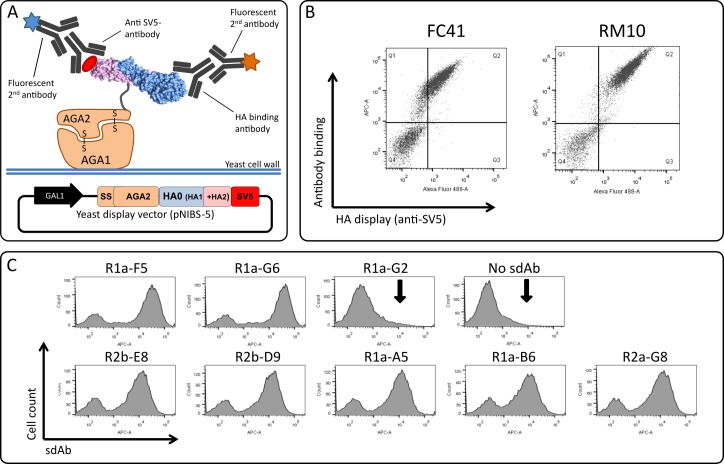
Display of hemagglutinin on yeast and evaluation of binding of single domain antibodies. (A) Schematic of the yeast display vector pNIBS-5. HA0 is full length hemagglutinin gene from A(H1N1) pdm09 comprising HA1 (blue) and HA2 (violet) domains fused in frame with yeast cell surface anchor protein AGA2 and a SV5 epitope tag. Expression and display is mediated by aga signal sequence (ss) and a galactose inducible promotor (GAL1). Display of HA0 is detected using a anti-SV5 monoclonal antibody (mAb). (B) Detection of A(H1N1)pdm09 HA0 display. Display and correct folding of full-length HA0 was confirmed by co-staining of yeast cells with an anti-SV5 mAb and the conformational specific IgG antibodies FC41 or RM10 control antibodies which bind to conformational epitopes in the HA stem or head domain respectively. (C) FACS plots of seven HA specific single domain antibodies (sdAbs) R1a-F5, R1a-G6, R2b-E8, R2b-D9, R1a-A5, R1a-B6, R2a-G8 [8] binding to yeast displayed HA0. Negative controls sdAb R1a-G2 and no sdAb control are shown. The vertical arrow indicates absence of binding.

The precursor full length HA protein (HA0) is cleaved post-translationally *in vivo* into two subunits, HA1 and HA2, linked together by a disulphide bond which remain assemble to form mature HA on the viral surface [2]. We chose to display precursor hemagglutinin (HA0) from A(H1N1)pdm09 on yeast rather than displaying separate HA1 and HA2 domains and relying on the correct assembly of the two domains on the yeast cell surface. Although the display of multi-chain proteins on yeast has been demonstrated [50] it is know that the HA2 domain does not fold correctly when expressed in the absence of the HA1 domain [56]. We sub-cloned a single open reading frame corresponding to the HA0 gene (residues HA1,1–329 to HA2,1–184, H3 numbering [3]) from A(H1N1)pdm09 into the yeast display vector pNIBS-5 fused to the C-terminus of the cell surface anchor protein Aga2p. A SV5 epitope tag was included at the C-terminal end of HA in order to detect full-length expression on the yeast cell surface simultaneously with antibody binding (Fig 1A). Yeast cells were separately labelled with an anti-SV5 antibody and two known conformational specific antibodies [20], to confirm the correct display and folding of HA. The stem binding antibody FC41 and the head binding antibody RM10 showed clear immunoreactivity to A(H1N1)pdm09 HA0 (Fig 1B), indicating that correctly folded head and stem domains were displayed on the yeast cell surface. We have previously isolated a panel of five cross-neutralising sdAbs to pandemic influenza A(H1N1)pdm09 virus hemagglutinin (HA) and highly pathogenic avian influenza H5N1 [8]. All sdAbs tested showed clear binding to yeast cells displaying HA demonstrating that their respective epitopes were intact (Fig 1C, Table 1).

**Table 1:** Location of sdAb epitope to stem or head region of hemagglutinin.

Antibody	HI activity H1N1 (X-181)^1^	Antibody neutralisation^2^	YD H1N1^3^	YD H5N1^3^	YD H7N7^3^	YD h1.s5^3^	YD h5.s1^3^	YD h1.s7^3^	YD h7.s1^3^	Low-pH treated HA^4^	Heat-treated HA^5^	Head/stem Binder^6^
**FC41—control**	-	H1/H5	++	++	-	-	++	-	++	ND	ND	Stem
**RM10—control**	ND	H1	++	-	-	++	-	++	-	ND	ND	Head
**MIA-H7 -control**	ND	ND	-	-	+	-	-	-	+	ND	ND	Head
**R1a-F5**	+	H1	++	-	-	++	-	++	-	-	-	Head
**R1a-G6**	+	H1	++	-	-	++	-	++	-	+	-	Head
**R2b-D9**	-	H1/H5/H2	++	++	-	-	++	-	-	-	-	Stem
**R2a-G8**	-	H1/H5	++	+	-	-	++	-	-	-	-	Stem
**R1a-B6**	-	H1/H5/H9	++	++	-	-	++	-	-	-	-	Stem
**R1a-A5**	-	H1/H5	++	++	-	-	++	-	-	-	-	Stem
**R2b-E8**	-	H1/H5	++	+	-	-	++	-	-	-	-	Stem

^1^ HI activity. Hemagglutination inhibition (HI) assay using laboratory adapted H1N1 strain (X-181). ND is not determined

^2^ Antibody neutralisation. H1N1, laboratory adapted X-181 strain (corresponding to A/California/07/2009), H5N1, NIBRG-14 (reverse genetics re-assortant of A/Vietnam/1194/2004), H9N2, NIBRG-91 (reverse genetics re-assortant of A/chicken/Hong Kong/G9/1997) and H2N2, NIBRG-147 (reverse genetics re-assortant of A/Singapore/1/1957). Antibodies FC41 and RM10 were whole IgG control antibodies [20].

^3^ Binding to yeast displayed (YD) wild type and chimeric HA. The mean fluorescence intensity (MFI) value of each antibody-chimera combination was divided by the value of wild-type H1N1 HA incubation, and the resulting ratio normalized to percentage values. Relative binding of sdAbs to each chimeric HA was categorized as follows; ≤ 20% was considered no binding (-), between 20% and 40% as intermediate binding (+) and ≥ 40% as strong binding (++) (S1 Fig)

^4^ Indicates if antibody binding was retained (+) or lost (-) following low pH treatment of HA.

^5^ Indicates if antibody binding was lost following heat treatment of HA displayed on yeast.

^6^ Prediction if antibody binds to head or stem region of HA.

The ability to neutralise virus and inhibit hemagglutination (HI positive) of red blood cells are an indication of blocking the receptor binding site within the head domain, whereas antibodies which neutralise virus, but are negative for HI, are predicted to bind to the stem region and neutralise virus via post-attachment mechanisms [9, 15]. Based on this assumption we predicted that sdAbs R2b-D9, R2a-G8, R1a-B6, R1a-A5 and R2b-E8 bind to the stem region whereas sdAbs R1a-F5 and R1a-G6 bind to the head domain and block receptor binding. To further de-lineate antibody binding we have used chimeric HAs comprising domains from different viral subtypes [57, 58], confirming head/stem discrimination of binding (Table 1, S1 Fig, S1 Supporting Information).

It has been previously showed that many of the human monoclonal antibodies with broad neutralizing activity function by blocking the low pH induced conformational changes in the HA stem so inhibiting viral membrane fusion [2, 22]. We compared the pH dependent binding of our sdAbs using HA incubated under conditions which mimics this conformational change (S2A–S2C Fig). We sub-cloned the head binding antibody R1a-F5 into the yeast display vector to capture pH-treated HA from A (H1N1)pdm09 viral antigen standard treated with different pH buffers. Antibodies predicted to bind to the stem region from our binding analysis using chimeric HA lost binding at low pH which was consistent with binding to a pH sensitive epitope in the stem region. The known head binding antibody R1a-G6 bound HA at both pH 4.8 and pH 8.0 equally well (S2C Fig) as expected. Antibody R1a-F5 retained binding at low pH which was also consistent with binding to the head domain, however the signal was much lower than R1a-G6. This can be explained by R1a-F5 being used as both capture antibody displayed on yeast and detecting antibody, limiting the analysis to trimeric forms of HA and excluding detection of the monomeric species.

As yeast is a robust organism resistant to a range of conditions, it is possible to carry out simple screening tests for stability using yeast displayed HA and flow cytometry, without the need to express and purify the antigen. Our panel of A(H1N1)pdm09 specific sdAbs were evaluated using heat-treatment of yeast cells and all sdAbs lost binding demonstrating that they are conformational specific, and so may be suitable for assessing HA stability. The loss of binding was not due to loss of HA at the cell surface as equivalent levels of antigen could be detected using the SV tag after heat treatment (S2D Fig). Yeast display offers a rapid, simple and efficient means to explore the specificity, stability, folding and mutational tolerance of HA under a range of conditions, some of which may be relevant to vaccine manufacturing.

### Antibodies R1a-G6 and R1a-F5 map to epitopes in the HA1 head domain

**Fig 2:**
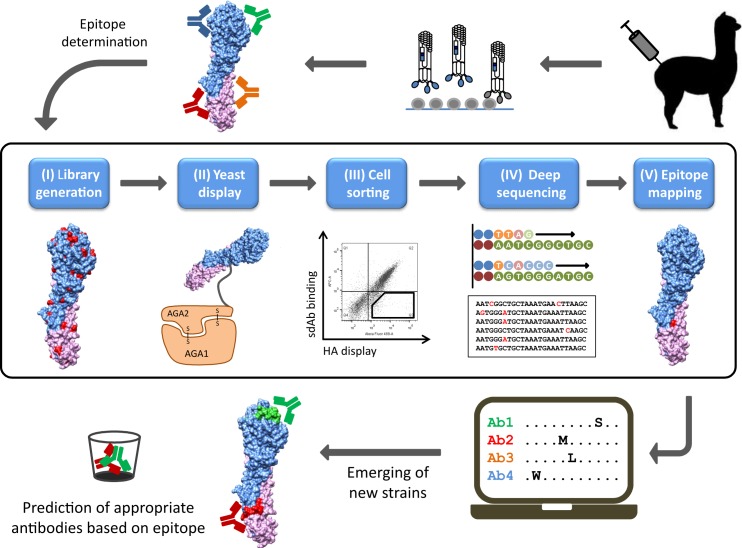
A general strategy for high-throughput epitope mapping of single domain antibodies to hemagglutinin. Generation of panels of high affinity monoclonal single domain antibodies (sdAbs or nanobodies) to hemagglutinin using immunisation of alpacas with HA and phage display technology, (i) design of a library of HA variants, (ii) display library on yeast cell surface, (iii) selection using flow cytometric cell sorting to enrich hemagglutinin variants that lose binding to sdAbs but retain display of correctly folded HA on yeast cell surface, (iv) pools of enriched HA mutants are then analysed using deep sequencing, (v) mutations are enriched and their frequencies in the selected population relative to the non-selected population are identified using bioinformatic analysis. Functional loss of binding is experimentally determined to confirm residues are energetically important and contribute to the antibody epitope. This approach can be used to generate a database of epitopes corresponding to diverse collections of sdAbs specific for HA, which upon the emergence of a new viral strain can be used to predict which antibodies could be chosen as suitable binding reagents for applications in diagnosis, research, immune surveillance and vaccine potency testing.

Our overall strategy to precisely map antibody epitopes to influenza HA is underpinned by yeast display and deep mutational scanning (Fig 2) [45, 46]. We have used random mutagenesis by error prone PCR to generate a yeast library of 2.25x10^7^ HA0 mutants. Low-error rate mutagenesis was used to bias our library towards single-point mutations. This limits the recovery of clones with multiple unrelated mutations, or mutations associated with HA misfolding, which would lead to the loss of display on the yeast cell surface and low library diversity.

**Fig 3:**
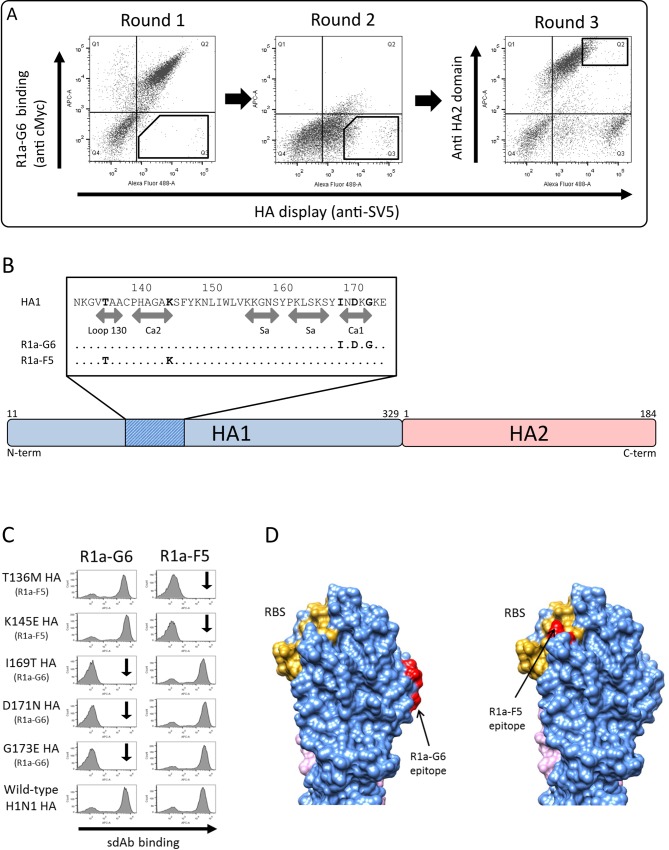
Mapping of antibody epitopes using conventional sequence analysis of a HA library selected with single domain antibodies binding the head domain. (A) FACS plots showing the initial HA library and following outputs incubated with 100 nM of R1a-G6 (round 1 and 2) and 100 nM of R1a-B6 (round 3). HA display (anti-SV5) is shown on the x-axis and R1a-G6 antibody binding (anti-cMyc tag) is shown on y-axis. For each round the gated population for cell sorting is shown. (B) Summary analysis of epitopes of sdAbs R1a-F5 and R1a-G6 located to the head domain (HA1). For each antibody epitope, the key residues are shown in bold. The position of antibody epitopes relative to loop 130, Ca2, Ca1 and Sa antigenic sites are indicated [3]. Specific mutations identified are given in S1 Table. (C) Example flow cytometry histograms plots of sdAbs R1a-G6 and R1a-F5 binding mutant and wild-type A(H1N1)pdm09 HAs displayed on yeast cells isolated from cell sorting (the sdAb used for the isolation of the specific HA mutants is indicated in parenthesis). The vertical arrow indicates no binding as expected relative to mutants isolated using the specific sdAb. (D) Surface structure of hemagglutinin HA from A(H1N1)pdm09 (PDB structure 3AL4) showing the HA1 domain (blue) and HA2 domain (violet). Receptor binding site (RBS) is indicated in yellow and key residues comprising the epitopes of R1a-G6 and R1a-F5 are shown in red.

To establish experimental sorting parameters and evaluate the suitability of the yeast displayed library, we initially focussed on mapping the epitopes of sdAbs predicted to bind to the head domain (Table 1, S1 Fig). Yeast cells displaying the library were incubated with anti-SV5 and purified R1a-G6, followed by sorting for both HA display and loss of antibody binding. A high antibody concentration of 100nM was used to maximise the recovery of all HA mutants which had completely lost binding. For the first round of sorting we collected 10,000 cells and the output was further enriched with a second round of cell sorting, maintaining a constant antibody concentration. To bias our selections towards mutations of energetically important residues and against allosteric mutations, which may influence sdAb binding at a residue distant from the epitope, we carried out a final round of selection using a non-competing stem binding antibody (Fig 3A). Thirty clones were randomly picked, sequenced and aligned to wild-type HA0 to identify candidate mutations (S1 Table). Clones containing multiple mutations, or mutations introducing/replacing cysteine or proline residues, were not tested further as they could be predicted to have indirect effects on antibody binding. Clones carrying single point mutations at three positions, Ile^169^, Asp^171^ and Gly^173^ (Fig 3B and 3D, S1 Table) within the antigenic site Ca1 [3] were enriched and loss of binding of R1a-G6 was confirmed (Fig 3C). The same strategy was applied for R1a-F5 and we identified mutations at Thr^136^ and Lys^145^ as disrupting of R1a-F5 antibody binding (Fig 3C). The epitope of R1a-F5 maps to the receptor binding site overlapping loop 130 and the antigenic site Ca2 (Fig 3B and 3D).

### Mutational scanning using deep sequencing predicts sdAb epitopes in the HA stem region

**Fig 4:**
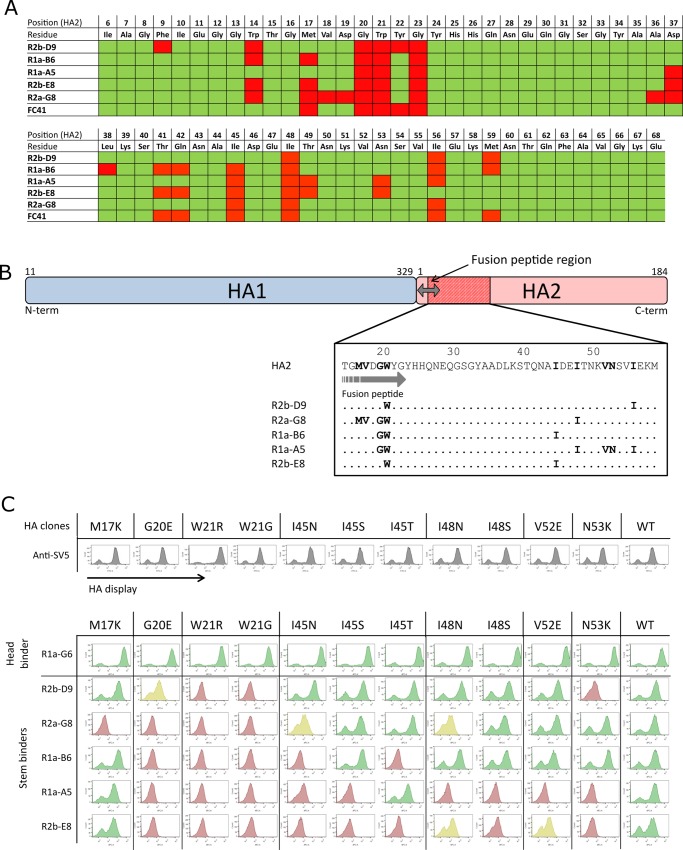
Mapping of antibody epitopes using deep sequence analysis of a HA library. (A) The table highlights residues predicted to be involved in sdAb binding and residues which have a minimal effect on antibody binding. Deep sequencing data from a single round of sorting were analysed and mutations identified. The enrichment factors for single amino acid mutations were calculated as the ratio of a given mutation after selection relative to its ratio prior to selection. Positions showing mutations which were highly enriched (E≥5x) were identified as mutational ‘hotspots’ (red squares) and predicted to have a direct involvement in antibody binding, whereas positions which did not yield any enriched mutations (E<5x) were predicted to have little role in antibody binding (green squares). The epitope map shows mutational hotspots and coldspots within the region Ile^6^ and Glu^68^ spanning the HA2 domain (region HA1-Gly^303^ to HA2-Ala^5^, which gave only ‘coldspots’ except R329G for R1a-B6, is not included in Fig 4A. Detailed listing of all mutations is given in S2 Table. Analysis of the unselected library showed all residues were mutated to between 3 and 8 different amino acids (S4 Table). Sequencing datasets are available for download through accession number PRJEB15301. (B) Highly enriched mutated residues after three rounds of selection are shown in relation to the HA2 domain and fusion peptide for sdAbs R2b-D9, R1a-B6, R2a-G8, R1a-A5, R2b-E8. The fusion peptide is shown with grey arrow (HA2-Gly^1^ to HA2-Gly^23^, H3 numbering). For each antibody epitope, the key residues were reported in bold and residues where antibody binding was unaffected by mutation are shown as dots. (C) Mutant HA genes carrying single amino acid mutations at seven different positions predicted by deep sequencing were tested experimentally to confirm their role in antibody binding. Flow cytometry histograms are shown for antibody binding to wild-type (WT) H1N1 HA and each of the single point mutations indicated within the HA2 domain. The sdAbs are grouped as head-binding (R1a-G6) and stem-binding (R2b-D9, R2a-G8, R1a-B6, R1a-A5, R2b-E8). Mutations that eliminate antibody binding are shown in red, those that reduce binding but do not completely eliminate it are shown in yellow and those that have no effect on binding are shown in green. Each mutations was shown to have no effect on HA display (grey histogram). We determined the extent of antibody binding as follows; the MFI value of each antibody-mutant pair was divided by the value of the wild-type H1N1 HA incubation, and the resulting ratio normalized to percentage values. Relative binding of sdAbs to each displayed mutant was categorized as follows; ≤20% no binding (red), between 20% and 40% intermediate binding (yellow) and ≥40% strong binding (green).

For putative stem binding sdAbs (Table 1) we have extended our analysis by using deep sequencing of the entire population of HA mutations (Fig 4A). Our initial studies using conventional sequencing, identified Trp^21^ as a key residue within the HA stem which guided our deep sequence analysis to a single 294 nucleotide amplicon (HA1-Gly^303^– HA2-Asn^71^, H3 numbering) covering the C-terminal end of the HA1 domain and the N-terminal end of the HA2 domain. This amplicon overlaps Trp^21^ and the major functional components of the HA stem region including the fusion peptide. A single round of selection of the HA library was carried with each of the cross-neutralising sdAbs and analysed by deep sequencing. We excluded mutations that introduced/replaced cysteine or proline residues from our analysis as they are likely to have structural effects which are not directly linked to antibody binding. After ranking the cumulative frequency of each amino acid substitution in the total population and calculating the enrichment factor, we were able to predict residues crucial for binding (mutational ‘hotspots’) and residues which have a minimal effect on antibody binding (mutational ‘coldspots’). Mutations with frequency of greater than 0.5% (f≥0.5%) and a enrichment factor of greater than 5 (enrichment E≥5x) were classified as mutational ‘hotspots’ and predicted to have a direct involvement in antibody binding (Fig 4A). Within the 98 amino acid region analysed by deep sequencing, 20 residues were identified as having a potential impact on antibody binding. The remaining residues within this region did not give any interfering mutations and were predicted to be sites with a minimal role in binding. Analysis of the unselected library showed that all residues within Gly^303^-Asn^71^ were mutated to between 3 and 8 different amino acids, so each site was taken as being tested for its involvement in binding (S2 Table). Selections on all cross-neutralising sdAbs gave similar mutational fingerprints which was consistent with their having overlapping epitopes centred around the key Trp^21^ residue. However there were clear differences in mutations enriched between different sdAbs reflecting more subtle differences in their respective epitopes. The majority of residues identified as hotspots were enriched as multiple mutations of the same residue (e.g. M17V/M17R/M17K, I45F/I45A/I45N/I45S/I45T, I48T/I48N/I48S, G20R/G20E, W21R/W21G, N53I/N53K/N53S/N53T) (S2 Table) reflecting the particular importance of these residues for each sdAbs.

After three rounds of selection the spectrum of mutations reduced to a smaller number of positions with the greatest enrichment factors (S2 Table, Fig 4B). Mutations predicted by deep sequencing to directly impact antibody binding were subsequently tested experimentally. One of the advantages of yeast display is that HA mutations can be constructed in a matter of days and screened for binding against a wide panel of antibodies without the need to purify HA antigen. In total seven different positions (Met^17^, Gly^20^, Trp^21^, Ile^45^, Ile^48^, Val^52^ and Asn^53^) carrying 11 different mutations were tested. Selected yeast clones were first checked for display, assessing that the single amino acid changes do not affect HA surface expression, and then labelled with the panel of sdAbs including the head binding control antibody R1a-G6 (Fig 4C). The loss or reduction in antibody binding agreed with the predictions made by deep sequencing (Fig 4A and 4B). Mutation of the highly conserved Trp^21^ to both an arginine or glycine residue completely abolished the binding of all sdAbs, whilst binding of the head binders R1a-F5 and R1a-G6 remained unaffected indicating that the mutated HA can still be processed through the yeast secretion machinery and displayed as a folded protein. The residue Trp^21^ lies at the C-terminal end of the fusion peptide, which is intimately involved in viral membrane fusion. This position within the sdAb epitope is consistent with their losing binding at low pH which leads to major structural rearrangements of the fusion peptide (S2B Fig) [59]. The adjacent residue Gly^20^ when mutated to glutamate eliminates binding to the sdAbs (R2a-G8, R1a-B6, R1a-A5, R2b-E8) whilst having only a partial effect on R2b-D9. Both Gly^20^ and Trp^21^ are within the fusion peptide and are absolutely conserved across all group 1 viral subtypes reflecting their functional importance.

Although all cross-neutralising sdAbs recognised this highly conserved region in the stem region, we observed differences in each antibodies epitope footprint. For example, Ile^45^, which is located adjacent to the key Trp^21^ in the HA structure was shown to affect binding of R2a-G8, R1a-B6, R1a-A5 and R2b-E8, whereas antibody R2b-D9 remained unaffected by mutation at this site. In addition mutations at residue Ile^48^ only affected binding of R1a-A5, R2b-E8 and to a lesser extent R2a-G8. Both N53K and V52E mutations were shown to uniquely affect antibodies R1a-A5 and R2b-E8 whereas the binding of other antibodies were tolerant of these mutations (Fig 4C). Antibody R2a-G8 was uniquely affected by the M17K mutation whereas other antibodies remained tolerant of this change. Additionally, the adjacent mutations V18A and D19V/G/E were predicted by mutational scanning to uniquely affect R2a-G8 binding however these mutations were not experimentally confirmed (Fig 4A, S2 Table). This suggests the epitope of R2a-G8 although overlapping is distinct to the other antibodies in having a significantly larger epitope footprint.

### Rational design and testing of naturally occurring substitutions within HA stem epitope of A(H1N1)pdm09

**Fig 5:**
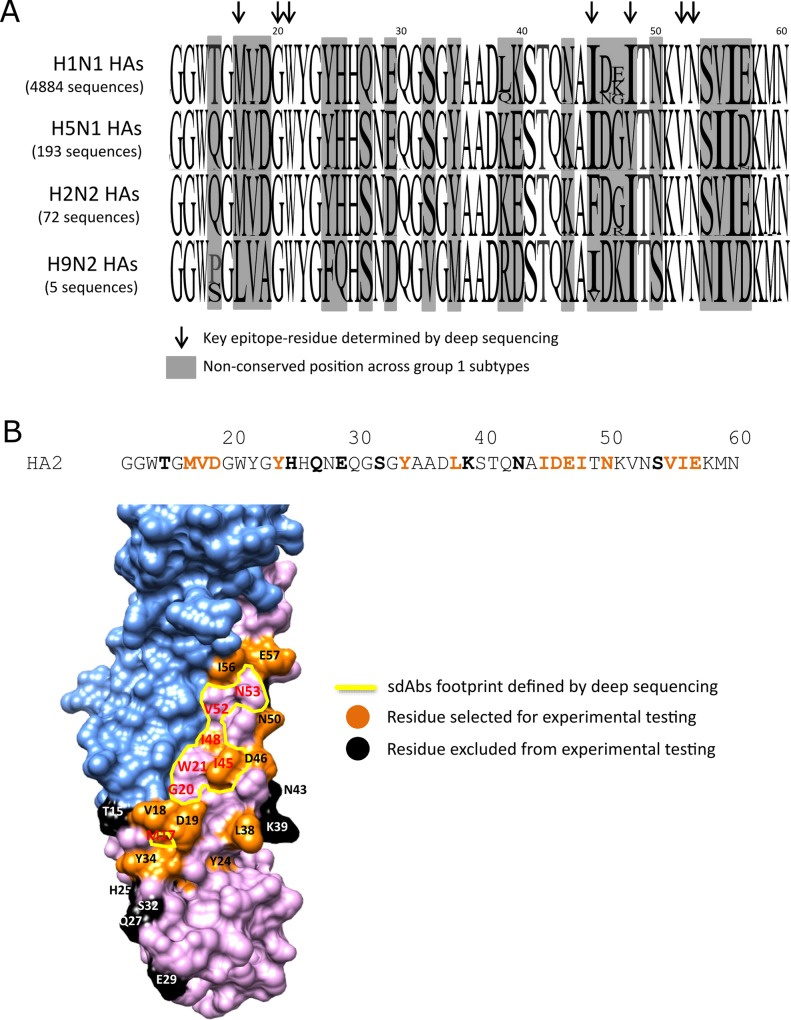
R1a-B6 key residues on Hemagglutinin A(H1N1)pdm09 crystal structure. (A) A total of 5151 full-length HA sequences corresponding to H1N1, H5N1, H2N2 and H9N2 viral subtypes were aligned and the relative diversity at non-conserved positions was evaluated and showed as a logo sequence (S3 Table). Alignment of HA2 Gly^12^-Asn^60^ is shown. Residues predicted to form part of the epitope footprint of our stem binding sdAb panel and identified by yeast display and deep sequencing indicated by black arrows (Fig 5B and 5C). Non-conserved residues either within subtype or across subtypes are highlighted by grey boxes. (B) Residues that show diversity but are buried in the HA structure, positioned on the reverse face of the HA monomer or at the interface of a HA trimer were not considered for mutagenesis and testing (black residues) (S3 Table). Residues that vary across viral subtypes are surface exposed and close in the structure to the binding footprint defined by deep sequencing and to residues tested in Fig 5 (residues highlighted in red) were chosen for experimental testing (orange residues).

**Fig 6:**
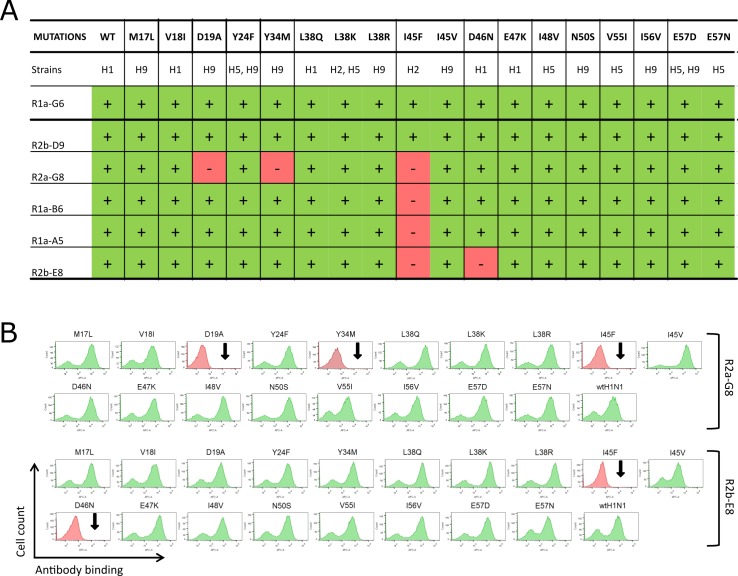
Experimental testing of naturally occurring mutations within epitope footprint. (A) Table showing binding activity of sdAbs to a panel of HA mutants carrying naturally occurring mutations. The extent of antibody binding was determined by dividing the MFI value of each antibody-mutant pair by the value of the wild-type H1N1 HA incubation, and the resulting ratio normalized to percentage values. Relative binding of sdAbs to each displayed mutant were categorized as no binding (-, red) and strong binding (+, green). (B) Flow cytometry histograms showing binding of R2a-G8 and R2b-E8 to wild-type H1N1 and panel of HA mutants. Absence of binding for D19A,Y34M, I45F and D46N mutation are indicated with a black arrow.

To further refine the epitope map of our stem binding sdAbs, we designed a small panel of naturally occurring mutations covering differences between HA subtypes. We aligned 4881 H1N1 193 H5N1, 72 H2N2 and 5 H9N2 full-length hemagglutinin sequences (www.fludb.com) and the amino acid antigenic diversity of the first 60 residues of HA2 domain (Gly^1^ –Asn^60^) were summarized as a sequence logo (Fig 5A). We identified 22 positions showing a variability of two or more residues, either between subtypes or within a single subtype. After mapping each position on the hemagglutinin structure relative to the epitope defined by deep sequencing (Fig 4C), we identified those residues with the potential to make direct contact with the antibody paratope. As such we did not consider residues buried in the HA structure, those residues distant from the highly conserved Trp^21^, or positioned on the reverse face of the HA monomer (S3 Table, Fig 5B). Using these criteria, residues Met^17^, Val^18^, Asp^19^, Tyr^24^, Tyr^34^, Leu^38^, Iso^45^, Asp^46^, Glu^47^, Ile^48^, Asn^50^, Val^55^, Ile^56^ and Glu^57^ were chosen as eighteen different mutations for experimental testing (Fig 6).

**Fig 7:**
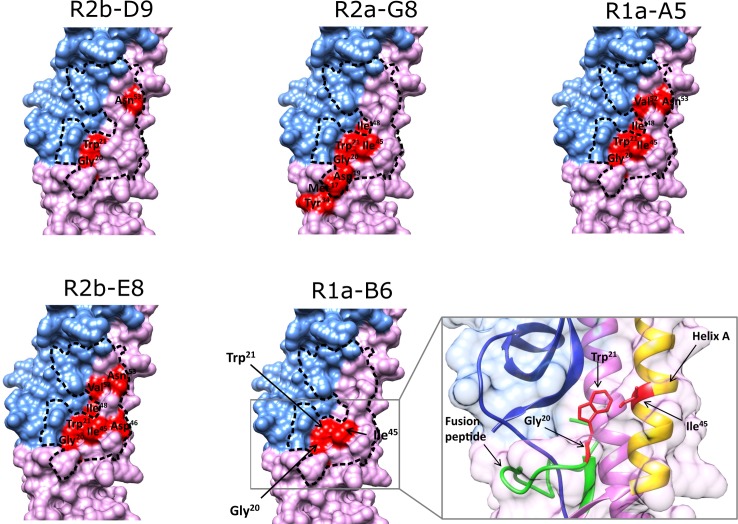
Relative binding footprint of individual within the HA stem epitope. Surface structure models of hemagglutinin (HA) A(H1N1)pdm09 (PDB structure 3AL4) showing the two domains (HA1 in blue, HA2 in violet) and the key epitope residues of each sdAb. The key epitope residues of human antibody CR6261, defined by X-ray crystallography [22], is indicated with a dotted black line and demonstrates overlapping sdAb epitopes. The epitope footprint of each sdAb is shown in red relative to the HA stem and combines residues identified by deep mutational scanning (Fig 5) and rational mutagenesis with naturally occurring subtype specific substitutions (Fig 7). The final panel shows a exploded view of the R1a-B6 epitope with key Gly^20^, Trp^21^ and Ile^45^ residues shown in red. Helix A and fusion peptide are highlighted in yellow and green respectively.

What was immediately striking was that very few naturally occurring substitutions affected sdAb however some notable sub-type specific mutations could be correlated with the cross-reactivity profile of individual antibodies. For example the I45F mutation correlates with H2 viral subtypes (S3 Table) and this was shown to eliminate binding of all stem binding sdAbs except R2b-D9 (Fig 6). This was in agreement with the mutational scanning analysis which showed that Ile^45^ mutations uniquely did not impact R2b-D9 suggesting that this antibody was able to tolerate this H2 specific change or this residue was outside the epitope footprint for R2b-D9 (Fig 4A–4C). This is in agreement with our previous analysis which demonstrates that R2b-D9 was distinct amongst our panel of antibodies in that its cross-neutralising activity included H2N2 virus as a monovalent antibody [8]. All experimentally confirmed positions were mapped onto the surface structure of A(H1N1)pdm09 HA to compare relative epitope footprints of each sdAbs and also the extent of overlap with the human monoclonal antibody CR6261 [20] (Fig 7).

The D46N mutation was shown to uniquely interfere with R2b-E8 binding whereas all other stem binding antibodies were unaffected (Fig 6A). Binding specificity to D46N in the context of the entire HA sequence from different H1N1 and H5N1 viral strains was subsequently tested using surface plasmon resonance (SPR) (Table 2, S4 Fig). R2b-E8 specificity was shown to correlate with D46 in HA genes from 1918 H1N1 pandemic (Spanish flu), the antigenically related 2009 H1N1 pandemic (swine flu) and highly pathogenic H5N1 (avian influenza) virus whereas binding was absent in seasonal H1N1 strains carrying N46. This confirms D46N as a key determinant of R2b-E8 specificity and demonstrates its potential utility in differentiating strains derived from A(H1N1)pdm09 from other circulating seasonal H1N1 strains (S3 Fig, Table 2). The 2009 H1N1 virus continues to evolve and a new prominent substitution in the HA2 domain, E47K, has emerged in 2010 which is located behind D46N in the HA structure (Fig 5B). The HA2-E47K substitution has been shown to correlate with a lower threshold pH for viral membrane fusion which enhances viral infectivity and stability [60]. This substitution was recreated as a yeast displayed product and antibodies R2b-D9, R2a-G8, R1a-A5, R1a-B6 and R2b-E8 were all shown to retain binding (Fig 6A and 6B). This was confirmed using SPR with equivalent binding seen on the drifted H1N1 strain A/Christchurch/16/2010 (HA2–K47) compared to the parental A/California/07/2009 (HA2-E47) (Table 2, S4 Fig). It is also interesting to note that E47 was identified as a mutational ‘coldspot’ using our deep mutational scanning (Fig 4A) and confirmation that HA2-K47 was present in the starting library (S3 Table) suggests that the preservation of binding to this drifted H1N1 strain binding could have been predicted a priori.

**Table 2:** Correlation of sdAb binding with the D46N mutation in H1N1 hemagglutinin strains between 1918 and 2010.

	A/SouthCarolina/1/1918(H1N1)–D46		A/NewCaledonia/20/1999(H1N1)–N46		A/SolomonIslands/3/2006(H1N1)–N46		A/Brisbane/57/2007(H1N1)–N46		A/California/07/2009(H1N1)pdm09 –D46		A/Christchurch/16/2010(H1N1)–D46		A/Vietnam/1194/2004(H5N1)–D46	
	*koff* (s^-1^)	K_D_ (nM)	*koff* (s^-1^)	K_D_ (nM)	*koff* (s^-1^)	K_D_ (nM)	*koff* (s^-1^)	K_D_ (nM)	*koff* (s^-1^)	K_D_ (nM)	*koff* (s^-1^)	K_D_ (nM)	*koff* (s^-1^)	K_D_ (nM)
**R2b-D9**	5.8x10^-5^	0.08*	4.4x10^-4^	0.10	3.0x10^-4^	0.35	9.0x10^-5^	0.18	7.6x10^-5^	0.13	4.2x10^-5^	0.05*	3.1x10^-5^	0.13
**R2a-G8**	1.9x10^-4^	0.16	8.3x10^-5^	0.07*	1.3x10^-4^	0.12	1.4x10^-4^	0.13	3.9x10^-4^	0.36	1.8x10^-4^	0.50	1.0x10^-3^	1.66
**R1a-B6**	2.6x10^-4^	0.23	1.8x10^-3^	1.82	2.6x10^-3^	2.45	2.2x10^-3^	2.84	1.8x10^-4^	0.21	1.2x10^-5^	0.23	5.2x10^-4^	1.15
**R1a-A5**	4.5x10^-4^	0.62	1.8x10^-3^	1.45	1.9x10^-3^	1.69	1.7x10^-3^	1.81	1.7x10^-4^	0.35	1.7x10^-4^	0.39	8.6x10^-5^	0.21
**R2b-E8**	5.1x10^-4^	0.64	-	-	1.3x10^-2^	374	-	-	3.5x10^-4^	0.96	2.4x10^-4^	0.65	1.6x10^-3^	4.80

Dissociation rate constant *koff*, equilibrium dissociation constants K_D_ determined by single cycle kinetics on a high density surface (approximately 5,000 RU) of H1N1 subtypes between 1918 and 2010 (A/South Carolina/1/18, A/New Caledonia/20/99, A/Solomon Islands/3/2006, A/Brisbane/57/2007, A/California/07/2009, A/Christchurch/16/2010) and H5N1 subtype A/Vietnam/1194/2004. The presence of either aspartate (D) or asparagine (N) at residue 46 of the HA2 domain (H3 numbering) is indicated for each HA antigen subtype. Analysis was carried out using BIAevaluation software and a 1:1 fitting model.–indicates no binding or kinetic data could be measured.

* indicates binding affinity is close to the limits that can be accurately measured using BIAevaluation software.

## Discussion

The response to the 2009 A(H1N1) influenza pandemic has highlighted the need for additional strategies of intervention which preclude the prior availability of the influenza strain. With this in mind we have previously identified a panel of five cross-neutralising single domain antibodies (nanobodies) to pandemic influenza A(H1N1)pdm09 virus hemagglutinin and highly pathogenic avian influenza H5N1 from an immune alpaca phage displayed library [8]. The focus of this study was to fully characterise where on HA they bind so as to understand their potential as universal binding tools and immunotherapeutics. Our approach has been to use yeast display in combination with deep sequencing to precisely map their epitopes and correlate this with the mechanism of action, cross-reactivity and potential resistance to antigenic escape. Rather than using conventional escape mutagenesis with live virus, which has limited utility for antibodies binding to functionally conserved residues in HA, we have used an experimental system where a yeast displayed library of randomly mutated HA molecules are selected for loss of binding to specific antibodies (Fig 2). This can be seen as a model to explore the evolution of influenza hemagglutinin in the presence of neutralising antibodies or other selective pressures, however crucially this approach is not constrained by the need to preserve viral infection. The only requirement is to maintain protein stability and the capacity to display correctly folded HA on the yeast cell surface. This means a more comprehensive scanning of the potential for mutational escape is possible and can be used to characterise antibodies which bind to functionally conserved epitopes in the HA stem.

Our initial studies had sought to group sdAbs as either head or stem binding based on activity in neutralisation assays, hemagglutination inhibition assays, binding profile on a series of chimeric HA and sensitivity of binding to low pH. We have defined two sdAbs, R1a-F5 and R1a-G6, as putative head binding antibodies and used yeast display to successfully demonstrate key contact residues in the head domain involved in binding. When mapped onto the structure of HA from A(H1N1)pdm09, the epitope of R1a-F5 was shown to overlap with the receptor binding site which is consistent with its activity in HI assays (Table 1). For both R1a-F5 and R1a-G6 the key residues comprising the epitope are not conserved across viral sub-types (data not shown) which is in agreement with their having limited H1N1 sub-type neutralising activity [8]. Mutants derived from R1a-G6 epitope mapping showed binding to R1a-F5-derived HA mutants and *vice versa*, demonstrating the distinct and non-overlapping nature of the epitopes of these two antibodies. This also indicates that the mutations identified result in precise disruption of the antibody binding site rather than more general structural effects (Fig 3). The use of yeast display to map antibody epitopes in the HA head domain has been described previously [42, 61] however the technology has not yet been successfully used to characterise antibodies binding outside the head nor were these studies conducted in combination with deep sequencing. We have used yeast display and deep mutational scanning for the first time to map cross-neutralising antibodies to the stem region and have shown cross-neutralising nanobodies (R1a-A5, R2b-E8, R2b-D9, R1a-B6, R2a-G8) bind to a highly conserved binding pocket. The use of deep sequencing has allowed a more comprehensive assessment of the mutational landscape through monitoring the mutagenesis of each amino acid position in terms of its enrichment or depletion without the need to maintain viral infection. Key mutational ‘hotspots’ were identified (Trp^14^, Met^17^, Gly^20^, Trp^21^, Gly^23^, Ileu^45^, Ileu^48^, Ileu^56^ and Met^59^), and comparison with publically available databases confirms that these residues are highly conserved across group 1 viral subtypes reflecting their functional importance. Other residues when mutated were not selectively enriched and as such represent mutational ‘coldspots’ with a low potential to impact antibody binding. We have further refined our antibody epitope map by rational protein design through testing several naturally occurring mutations which describe subtype variation within the region Gly^1^ –Asn^60^. These substitutions can be rapidly re-created in a yeast display format and tested for sdAb binding without the need to purify HA antigen. What was most striking was that the vast majority of these naturally occurring mutations did not impact sdAb binding (Fig 6). This contrasts with the much larger spectrum of residues predicted to impact antibody binding using random mutagenesis by error prone PCR which is only limited by selective pressure for folding and display and not influenced by the need to maintain viral infection. This suggests that this region represents a significant genetic barrier and is consistent with a low potential for the influenza virus to evolve HA mutations capable of escaping recognition by these sdAbs, highlighting their potential as universal binding reagents and immunotherapeutics.

By combining all mutations defined by deep mutational scanning and rational protein design, we have been able to compare the relative antibody footprints of each sdAb. Amongst the sdAbs R2b-D9 (Gly^20^, Trp^21^, Asn^53^) and R1a-B6 (Gly^20^, Trp^21^, Ileu^45^) were shown to have the lowest number of interfering mutations. Whereas for R2a-G8 (Met^17^, Asp^19^, Gly^20^, Trp^21^, Tyr^34^, Ileu^45^, Ile^48^), R1a-A5 (Gly^20^, Trp^21^, Ileu^45^, Ile^48^, Val^52^, Asn^53^) and R2b-E8 (Gly^20^, Trp^21^, Ile^45^, Asp^46^, Ile^48^, Val^52^, Asn^53^) there were a significantly greater number of mutations identified impacting antibody binding (Fig 7). This could be interpreted as R2b-D9 and R1a-B6 as presenting a higher potential genetic barrier for the virus to escape through natural processes of antigenic drift and shift due to their smaller antibody footprint. We speculate that this could translate into cross-reactivity being maintained over a longer period of time than might be possible for an antibody with a larger epitope footprint. It would be interesting to evaluate selected mutations in the context of a model of infection using, for example pseudotyped viruses [62], to investigate if these mutations interfere with infection and represent viable evolutionary paths by which the virus could proceed to escape antibody neutralisation. We also note that despite having a smaller footprint compared to other cross-neutralising antibodies antibody, R2b-D9 has the highest affinity (Table 2), with the broadest cross-neutralising activity against H1, H5 and H2 viral subtypes. The epitope footprint of R1a-B6 encompasses Trp^21^, Gly^20^ and Ile^45^. Mapping onto the HA structure showed that Trp^21^ and Gly^20^ are located at the C-terminal end of the fusion peptide, and Ile^45^ lies within the helix A of HA2 (Fig 7). This is consistent with these antibodies neutralising virus post–viral attachment and inhibiting low pH induced membrane fusion. The antibody footprint of R1a-B6 falls into a highly conserved hydrophobic pocket formed by residues of the fusion peptide, strands of HA1 domain and helix A of HA2 domain. Trp^21^ has also been defined as being important for interactions with the group 1 cross-reactive human antibodies F10 and CR6261, where a crucial interaction between the indole functional group of tryptophan and specific residues of human antibodies is a common feature [9, 22]. It has been hypothesised that F10 and CR6261 do not cross-react with group 2 HAs because of the orientation of the Trp^21^ side chain [9, 20], which may also explain the lack of binding of our panel of sdAbs to group 2 hemagglutinins H7 and H3 (data not shown). The nanobodies we have identified overlap with the epitopes of cross-neutralising human monoclonal antibodies (Fig 7), CR6261 and F10 which are both in clinical development as passive immunotherapeutics [23]. This demonstrates that immunisation of alpacas is an alternative route to high affinity cross-neutralising antibodies that could be expected to have similar potential as immunotherapeutics [63]. The utility of stem binding cross-neutralising nanobodies as immunotherapeutics can be explored by testing in an *in vivo* challenge model of H5N1 infection as has been described for other cross-neutralising antibodies in mice [64] and ferrets [17]. The high intrinsic stability and ability to withstand nebulisation are distinct advantages over human antibody formats and means that intra-nasal delivery is a real possibility [65] allowing deep penetration of nanobodies into the respiratory tract. Reformatting and optimisation of nanobodies to HA stem epitopes by humanisation [66] and incorporating effector functions through engineering nanobody Fc fusions [67, 68] will facilitate systemic delivery. This will give immediate short term passive immunity compared to the longer periods of time required to elicit a vaccine induced response. Longer term passive immunity with cross-neutralising human monoclonal antibodies can be achieved using adeno-associated viral (AAV) mediated gene delivery [69]. However this is complex and requires stable expression of two transgenes, equivalent expression of light chain and heavy chain plus their efficient association to form an antigen combining site. The cross-neutralising single domain nanobodies we have described in this study would represent a much simpler antibody format which could be expected to simplify some of the complexities of AAV mediated delivery of conventional antibodies.

The potential application of cross-reactive nanobodies to pandemic influenza extends beyond immune prophylaxis [17, 64], and includes serological surveillance [70] universal vaccine design [71] and vaccine potency standardisation [72, 73]. The development of cross-reactive monoclonal antibodies to assess structural integrity, quantity and clinical potency of HA in influenza vaccines is of particular interest [73–75]. Influenza viruses have a great capacity to change their genetic material and as such vaccine manufacturers have to update their vaccines every year to accommodate the new circulating strains. To test the potency of these vaccines and to ensure the correct antigen dose is administered, manufacturers need new calibrated reference serum from immunised sheep provided by essential regulatory laboratories [73]. The availability of universal reagents like the cross-reactive sdAbs reported in this study may eliminate the need to generate strain specific sheep antiserum which can take up to 6 weeks. In the context of the total 6 month vaccine development pipeline process from strain selection to vaccine release, a reduction of a few weeks in providing essential regulatory reagents is significant and has the potential to not only improve seasonal vaccine manufacturing but enhance the ability to respond effectively to future influenza pandemics by stockpiling suitable antibody reagents. We have shown that precise mapping of antibody epitopes and comparison against natural antigen databases allow a predictive assessment of how likely a virus is to escape antibody binding. For example naturally occurring mutations at positions Trp^21^, Gly^20^ and Ile^45^ could be expected to impact the ability of R1a-B6 to bind virus whereas mutations at other sites could be tolerated. The ability to predict antibody binding has been exemplified by correlating the specificity of R2b-E8 with the D46N substitution across different viral strains. The binding of R2b-E8 was shown to correlate with a aspartate residue at position 46 of the HA genes from 1918 H1N1 ‘Spanish flu’ pandemic strain (A/South Carolina/1/1918), the antigenically related 2009 H1N1 pandemic strain (A/California/04/2009) and highly pathogenic avian influenza H5N1 (A/Vietnam/1194/2004). However binding was absent in seasonal H1N1 strains carrying N46. Given that D46 is positioned within such a crucial part of the HA stem region and is conserved across two pandemic H1N1 strains and highly pathogenic H5N1 leads us to speculate that it has some evolutionary significance. In addition proximity D46 to the E47K substitution [60] known to be associated with improved viral fitness warrants further investigation and the unique specificity of R2b-E8 may be a useful probe for such purposes. The influenza virus presents a constantly moving target and it would be interesting to explore if R2b-E8 could be similarly evolved to catch an escaping virus. By creating a library of yeast displayed R2b-E8 mutations it would be possible to see if R2b-E8 variants can be isolated to overcome the naturally occurring HA-N46 mutation and regain binding activity.

A database of cross-reactive nanobodies, corresponding epitopes, mutational ‘hotspots’ and mutational ‘coldspots’ may facilitate the early selection of binding reagents for vaccine manufactures independent of the prior availability of viral antigen. The caveat is that combinations of mutational changes outside the specific epitope may in some cases result in either local or quaternary structural changes which cannot be easily predicted. In addition it may be difficult to predict the impact any changes in glycosylation might have on epitope accessibility. However, this could be addressed by creating cocktails of carefully chosen nanobodies to non-overlapping epitopes, furthermore the ability to rapidly generate yeast displayed HA mutants from new antigenically drifted or shifted HA sequences in a matter of days without the need for protein purification could allow the experimental testing of antibody binding if required.

## Supporting Information

S1 Fig Chimeric hemagglutinins construction and testing. (A) Schematic diagram of chimeric HAs construction using H1N1, H5N1 and H7N7 as templates and swapping HA1 region Cys^52^-Cys^277^ [58]. Chimeric hemagglutinins were tested experimentally to confirm antibody binding. (B) Flow cytometry histograms of antibody binding to wild-type H1N1, H5N1, H7N7 HAs and chimeric HAs. Anti-SV5 flow cytometry histograms show the extent of protein displayed for each hemagglutinin. Yeast cells are stained with sdAbs. The MFI value of each antibody-HA pair was divided by the value of the wild-type H1N1 HA incubation, and the resulting ratio given as percentage values. Relative binding of antibodies to each hemagglutinin was categorized as follows; ≤20% no binding (red), between 20% and 40% intermediate binding (yellow) and ≥40% strong binding (green).
(TIF)

S2 Fig Characterization of single domain antibody binding. (A) Schematic diagram of the large conformational changes known to occur within the stem region of HA upon viral membrane fusion within the acidified endosomal compartment. Pre-fusion state is shown on the left and fusion active state is shown on right. The fusion peptide is shown in dark green [76]. (B) Graph of mean fluorescence intensity (MFI) for a panel of seven different sdAbs showing relative binding to the yeast displayed HA0 at pH 8.0 (grey bars) or pH4.8 (white bars) activity. Antibody R1a-G6 does not compete with R1a-F5 for HA binding (data not shown). (C) Example FACS plots of sdAbs R1a-A5 and R1a-G6. Vertical arrow indicates absence of binding. (D) Graph showing binding of sdAbs to yeast displayed HA following heat treatment.
(TIF)

S3 Fig Sequence alignment of N-term ends of HA2 domain (N-term, Gly^1^-Thr^60^). Full-length sequences of six hemagglutinin genes were downloaded from the Influenza Research Database (www.fludb.org); A/South Carolina/1/1918 (H1N1)(AF117241), A/New Caledonia/20/1999, (H1N1)(AY289929), A/Solomon Islands/03/2006 (H1N1)(EU100724), A/Brisbane/59/2007 (H1N1)(CY163560), A/California/07/2009 (H1N1)(CY121680), A/Vietnam/1194/2004 (H5N1)(EF541402). The sequence alignment shows D/N variability at position 46 in HA2, indicated by a black arrow.
(TIF)

S4 Fig Single cycle kinetics analysis of binding to recombinant HA. Binding and affinity analysis of cross-reactive sdAbs against recombinant HA using single cycle kinetics [55]. Each of the cross-reactive antibodies R1a-A5, R1a-B6, R2a-G8, R2b-E8, R2b-D9, were tested on recombinant antigens from (A) A/South Carolina/1/1918 (H1N1)(B) A/New Caledonia/20/1999 (H1N1) (C) A/Solomon Islands/03/2006 (H1N1) (D) A/Brisbane/57/2007 (H1N1) (E) A/California/04/2009 (H1N1)(F) A/Christchurch/16/2010 (H1N1) (G) A/Vietnam/1194/2004 (H5N1). The curves were generated with the sequential injection of sdAbs at 1nM, 2.5nM, 5nM, and 10nM. Analysis was using BIAevaluation software and data was fitted to a 1:1 binding model. Dotted lines represent fitted curves and coloured lines represent raw data measurements. Affinity constants are shown in Table 2.
(TIF)

S1 Supporting Information Construction and characterization of chimeric hemagglutinins. (DOCX)

S1 Table Summary of Sanger sequencing analysis of head binders R1a-G6 and R1a-F5. (DOCX)

S2 Table Frequency and enrichment factors of specific mutations identified from deep sequence analysis of library selections. (DOCX)

S3 Table List of HA2 positions showing residue diversity among group 1 sub-type viral strains (H1, H2, H5 and H9) within residue HA2 Gly^1^-Asn^60^. (DOCX)

S4 Table List of mutation in the unselected library identified by deep sequencing (HA1 Gly^303^-HA2 Asn^71^). (DOCX)
