# Supplementary figures and images for: Cross-Neutralising Nanobodies Bind to a Conserved Pocket in the Hemagglutinin Stem Region Identified Using Yeast Display and Deep Mutational Scanning
Source: PLoS One. 2016 Oct 14;11(10):e0164296. doi: 10.1371/journal.pone.0164296 (PMC5065140; doi:10.1371/journal.pone.0164296)

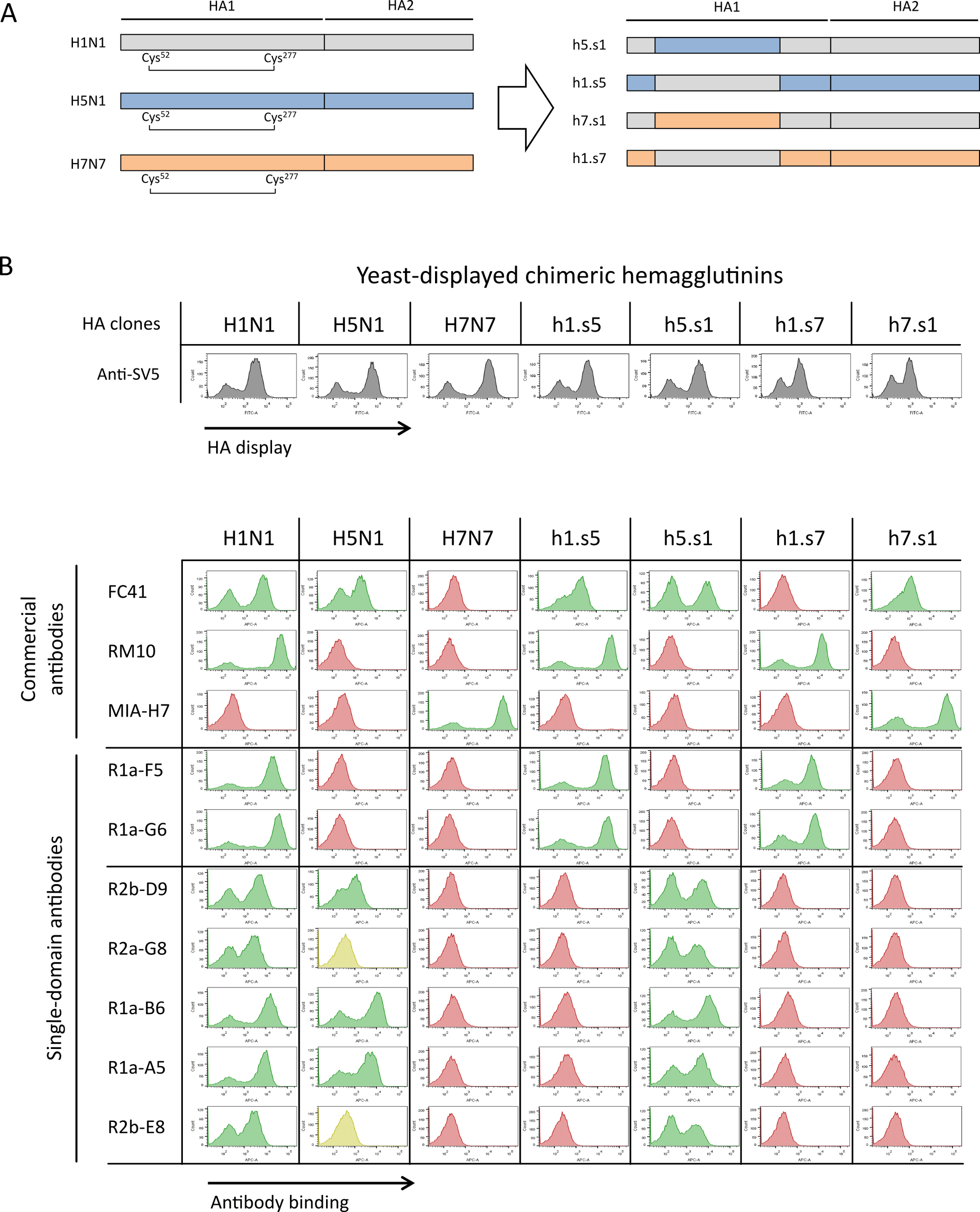

Supplement: S1 Fig — (A) Schematic diagram of chimeric HAs construction using H1N1, H5N1 and H7N7 as templates and swapping HA1 region Cys52-Cys277 [58]. Chimeric hemagglutinins were tested experimentally to confirm antibody binding. (B) Flow cytometry histograms of antibody binding to wild-type H1N1, H5N1, H7N7 HAs and chimeric HAs. Anti-SV5 flow cytometry histograms show the extent of protein displayed for each hemagglutinin. Yeast cells are stained with sdAbs. The MFI value of each antibody-HA pair was divided by the value of the wild-type H1N1 HA incubation, and the resulting ratio given as percentage values. Relative binding of antibodies to each hemagglutinin was categorized as follows; ≤20% no binding (red), between 20% and 40% intermediate binding (yellow) and ≥40% strong binding (green). (TIF) [file pone.0164296.s001.tif]

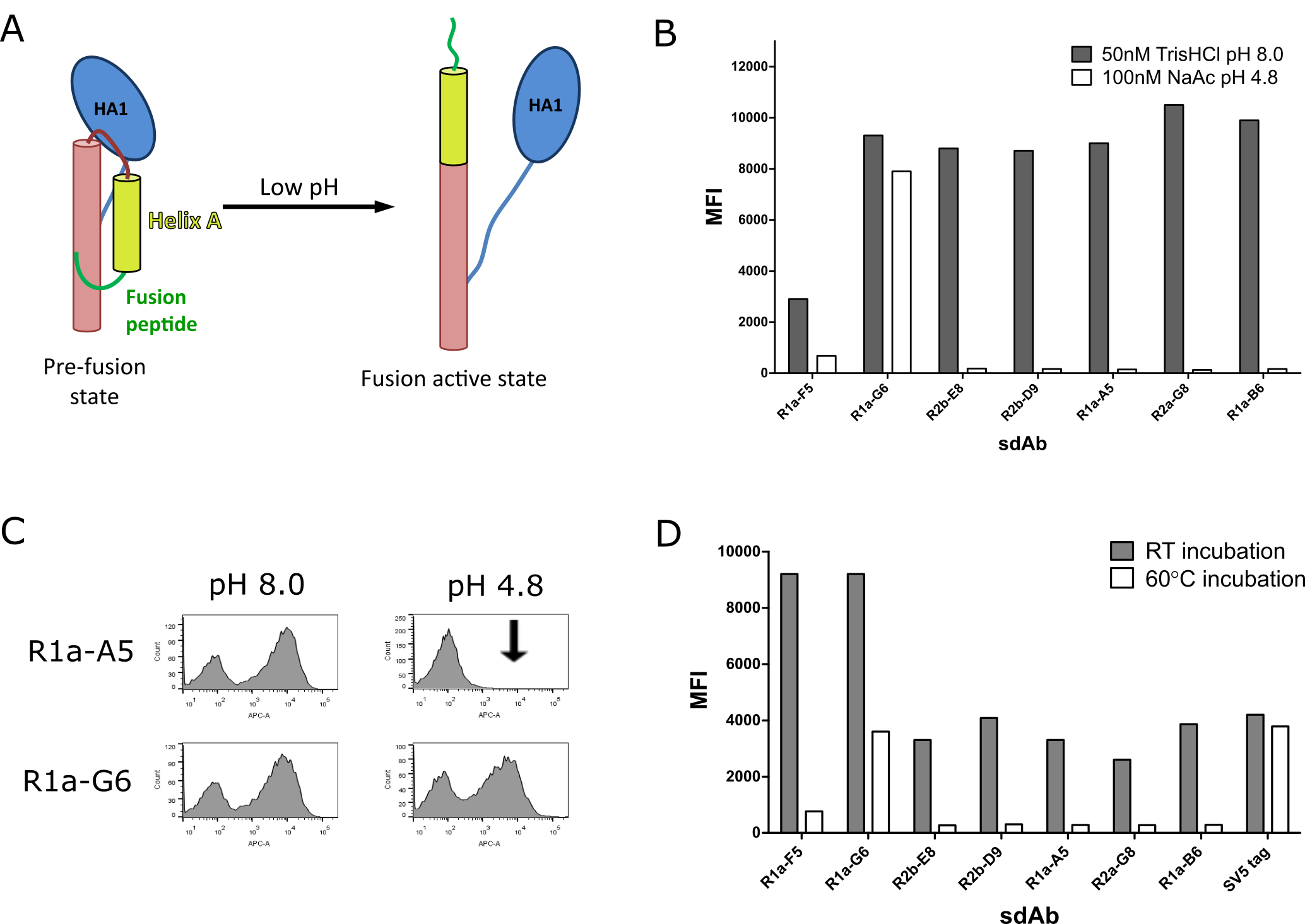

Supplement: S2 Fig — (A) Schematic diagram of the large conformational changes known to occur within the stem region of HA upon viral membrane fusion within the acidified endosomal compartment. Pre-fusion state is shown on the left and fusion active state is shown on right. The fusion peptide is shown in dark green [76]. (B) Graph of mean fluorescence intensity (MFI) for a panel of seven different sdAbs showing relative binding to the yeast displayed HA0 at pH 8.0 (grey bars) or pH4.8 (white bars) activity. Antibody R1a-G6 does not compete with R1a-F5 for HA binding (data not shown). (C) Example FACS plots of sdAbs R1a-A5 and R1a-G6. Vertical arrow indicates absence of binding. (D) Graph showing binding of sdAbs to yeast displayed HA following heat treatment. (TIF) [file pone.0164296.s002.tif]

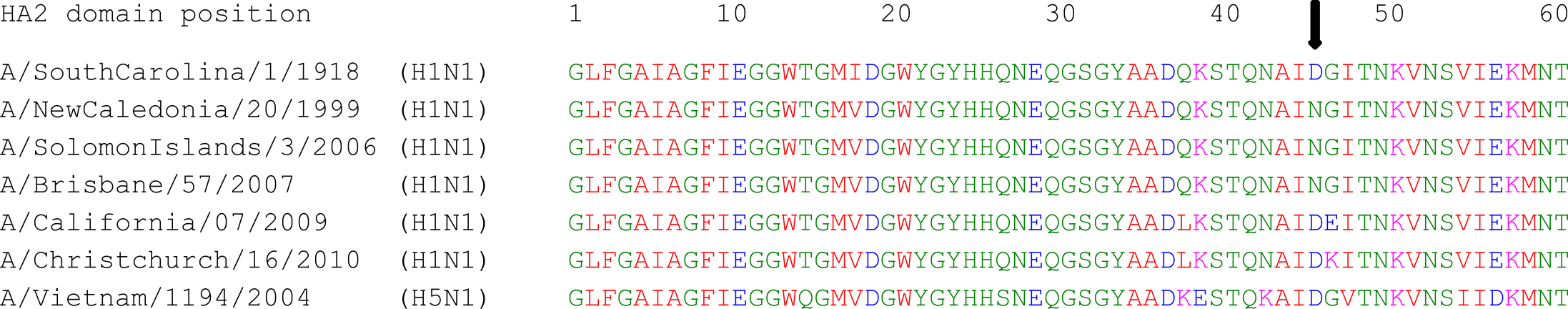

Supplement: S3 Fig — Full-length sequences of six hemagglutinin genes were downloaded from the Influenza Research Database (www.fludb.org); A/South Carolina/1/1918 (H1N1)(AF117241), A/New Caledonia/20/1999, (H1N1)(AY289929), A/Solomon Islands/03/2006 (H1N1)(EU100724), A/Brisbane/59/2007 (H1N1)(CY163560), A/California/07/2009 (H1N1)(CY121680), A/Vietnam/1194/2004 (H5N1)(EF541402). The sequence alignment shows D/N variability at position 46 in HA2, indicated by a black arrow. (TIF) [file pone.0164296.s003.tif]

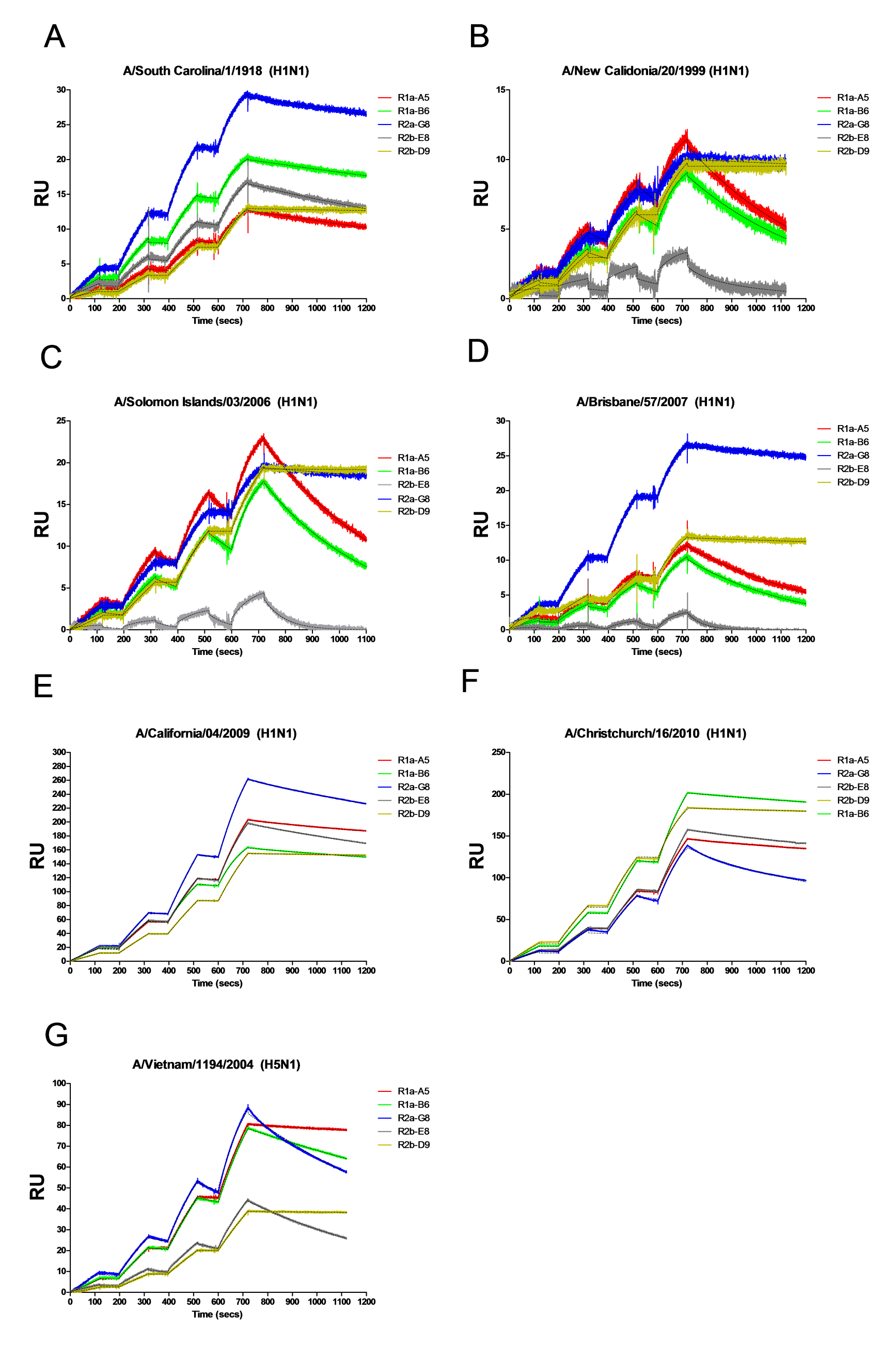

Supplement: S4 Fig — Binding and affinity analysis of cross-reactive sdAbs against recombinant HA using single cycle kinetics [55]. Each of the cross-reactive antibodies R1a-A5, R1a-B6, R2a-G8, R2b-E8, R2b-D9, were tested on recombinant antigens from (A) A/South Carolina/1/1918 (H1N1)(B) A/New Caledonia/20/1999 (H1N1) (C) A/Solomon Islands/03/2006 (H1N1) (D) A/Brisbane/57/2007 (H1N1) (E) A/California/04/2009 (H1N1)(F) A/Christchurch/16/2010 (H1N1) (G) A/Vietnam/1194/2004 (H5N1). The curves were generated with the sequential injection of sdAbs at 1nM, 2.5nM, 5nM, and 10nM. Analysis was using BIAevaluation software and data was fitted to a 1:1 binding model. Dotted lines represent fitted curves and coloured lines represent raw data measurements. Affinity constants are shown in Table 2. (TIF) [file pone.0164296.s004.tif]
