# Supplementary material for: Cross-Neutralising Nanobodies Bind to a Conserved Pocket in the Hemagglutinin Stem Region Identified Using Yeast Display and Deep Mutational Scanning
Source: PLoS One. 2016 Oct 14;11(10):e0164296. doi: 10.1371/journal.pone.0164296 (PMC5065140; doi:10.1371/journal.pone.0164296)
Supplement: S1 Supporting Information — (DOCX) [file pone.0164296.s005.docx]

S1 Supporting information

**Cross-neutralising nanobodies bind to a conserved pocket in the hemagglutinin stem region identified using yeast display and**

**deep mutational scanning**

**Tiziano Gaiotto and Simon E. Hufton**

## Construction of chimeric hemagglutinin antigens for yeast surface display

To obtain plasmids encoding chimeric hemagglutinins, i.e h1.s5, we amplified the H1N1-HA1 head domain (within Cys^52^-Cys^277^ region) through standard PCR amplification using oligonucleotides H1h-to H5s-For/H1h-toH5s-Rev, and H5N1 stalk domains (at the N- and C-terminus) through inverse PCR with oligonucleotides H5_iPCR-For/H5_iPCR-Rev. Overhanging sequences of the two fragments allowed recombination upon transformation into EBY100 yeast cells. The remaining chimeras were generated as follow; h5.s1 combining H5N1 head (H5h-toH1s-For/H5h-toH1s-Rev) and H1N1 stem domains (H1_iPCR-For/H1_iPCR-Rev), h1.s7 combining H1N1 head (H1h-toH7s-For/H1h-toH7s-Rev) and H7N7 stem domains (H7_iPCR-For/H7_iPCR-Rev), and h7.s1 combining H7N7 head (H7h-toH1s-For/H7h-toH1s-Rev) and H1N1 stem domains (H1_iPCR-For/H1_iPCR-Rev) (a list of oligonucleotide sequences available on request). Plasmids from single colonies were recovered and sequenced to check for the correct exchange of HA domains.

## Characterisation of sdAb binding to HA using yeast displayed chimeric HA antigens

Recent studies have shown that as the Cys^52^-Cys^277^ disulphide bond in the HA1 domain is broadly conserved, HA domains from different viral subtypes can be exchanged without disrupting the overall structure [1,2]. Chimeric constructs of head (HA1) and stalk domain (HA2) from group 1 viral subtypes A(H1N1)pdm09 and A/VietNam/1203/2004 (H5N1) plus the group 2 subtype A/Netherlands/219/2003 (H7N7) (Table 1, S1 Fig) were tested for display and antibody binding. Chimeras expressing the H1N1 head domain (h1.s5 and h1.s7) showed clear binding to the control conformational H1N1 specific head binding antibody RM10 proving that the H1 head domain was correctly folded and displayed on yeast. The control antibody FC41, which binds to a conformational epitope in the stem region [3], showed clear cross-reactivity with wild-type H1N1, H5N1 and binds the chimeric construct h5.s1 demonstrating that the stem region is likely to be displayed in an authentic conformation. Comparing the binding profile of commercial antibodies to our sdAbs, we found that R1a-G6 and R1a-F5 show similar binding profiles to RM10 and as such were predicted to bind to the head domain which was consistent with their being HI positive. Whereas R2b-D9, R2a-G8, R1a-B6, R1a-A5 and R2b-E8, show a similar binding profile to FC41 and were predicted to bind to epitopes in the HA stem region which was consistent with their being HI negative.

## Reference List

## 1. Hai R, Krammer F, Tan GS, Pica N, Eggink D, Maamary J, Margine I, Albrecht RA, Palese P (2012) Influenza viruses expressing chimeric hemagglutinins: globular head and stalk domains derived from different subtypes. J Virol 86: 5774-5781. JVI.00137-12 [pii];10.1128/JVI.00137-12 [doi].

## 2. Steel J, Lowen AC, Wang TT, Yondola M, Gao Q, Haye K, Garcia-Sastre A, Palese P (2010) Influenza virus vaccine based on the conserved hemagglutinin stalk domain. MBio 1. 10.1128/mBio.00018-10 [doi].

## 3. Corti D, Voss J, Gamblin SJ, Codoni G, Macagno A, Jarrossay D, Vachieri SG, Pinna D, Minola A, Vanzetta F, Silacci C, Fernandez-Rodriguez BM, Agatic G, Bianchi S, Giacchetto-Sasselli I, Calder L, Sallusto F, Collins P, Haire LF, Temperton N, Langedijk JP, Skehel JJ, Lanzavecchia A (2011) A neutralizing antibody selected from plasma cells that binds to group 1 and group 2 influenza A hemagglutinins. Science 333: 850-856. science.1205669 [pii];10.1126/science.1205669 [doi].
