# Supplementary material for: Cross-Neutralising Nanobodies Bind to a Conserved Pocket in the Hemagglutinin Stem Region Identified Using Yeast Display and Deep Mutational Scanning
Source: PLoS One. 2016 Oct 14;11(10):e0164296. doi: 10.1371/journal.pone.0164296 (PMC5065140; doi:10.1371/journal.pone.0164296)
Supplement: S1 Table — (DOCX) [file pone.0164296.s006.docx]

**S1 Table. Summary of Sanger sequencing analysis of head binders R1a-G6 and R1a-F5.**

| **R1a-G6 output analysis** | | **R1a-F5 output analysis** | |
| --- | --- | --- | --- |
|  | Frequency |  | Frequency |
| Sequences with good quality reads | 28/30 | Sequences with good quality reads | 30/30 |
| Single-residue mutants | 21/30 | Single-residue mutants | 10/30 |
| Cysteine/Proline mutants or with mutations in more than one position | 7/30 | Cysteine/Proline mutants or with mutations in more than one position | 19/30 |
| Mutations | # of mutants | Mutations | # of mutants |
| I169T | 14 | T136A | 1 |
| I169F | 2 | T136M | 1 |
| D171N | 1 | K145E | 4 |
| G173E | 3 | K145M | 3 |
| G173R | 1 | K145T | 1 |

Mutations are listed according to H3 numbering of HA1 domain.
