# Supplementary material for: Cross-Neutralising Nanobodies Bind to a Conserved Pocket in the Hemagglutinin Stem Region Identified Using Yeast Display and Deep Mutational Scanning
Source: PLoS One. 2016 Oct 14;11(10):e0164296. doi: 10.1371/journal.pone.0164296 (PMC5065140; doi:10.1371/journal.pone.0164296)
Supplement: S2 Table — (DOCX) [file pone.0164296.s007.docx]

**S2 Table. Frequency and enrichment factors of specific mutations identified from deep sequence analysis of library selections.**

| **R2b-D9 output analysis** | | | | |
| --- | --- | --- | --- | --- |
|  | Output I | | Output III | |
| Mutation | Frequency (F, %) | Enrichment factor (E) | Frequency (F, %) | Enrichment factor (E) |
| F9S | 0.58 | 5.61 | - | - |
| W14R | 1.26 | 6.51 | - | - |
| M17V | 0.52 | 4.62 | - | - |
| G20R | 1.01 | 37.11 | - | - |
| W21G | 0.95 | 19.34 | 4.00 | 81.60 |
| W21R | 8.24 | 50.34 | 48.21 | 294.57 |
| Y22H | 0.48 | 8.76 | - | - |
| G23D | 1.04 | 30.65 | - | - |
| G23S | 0.80 | 10.14 | - | - |
| I45T | 0.47 | 4.48 | - | - |
| I48T | 0.86 | 11.26 | - | - |
| I56T | 0.87 | 10.79 | 0.63 | 12.87 |
| M59T | 0.65 | 7.86 | - | - |

| **R1a-B6 output analysis** | | | | |
| --- | --- | --- | --- | --- |
|  | Output I | | Output III | |
| Mutation | Frequency (F, %) | Enrichment factor (E) | Frequency (F, %) | Enrichment factor (E) |
| W14R | 1.16 | 6.00 | - | - |
| M17V | 0.69 | 6.08 | - | - |
| R321G (HA1) | 0.64 | 5.08 | - | - |
| G20E | 1.07 | 34.71 | 2.11 | 68.31 |
| G20R | 0.78 | 28.83 | - | - |
| W21G | 0.95 | 19.47 | - | - |
| W21R | 6.04 | 36.92 | 26.21 | 160.15 |
| G23D | 0.71 | 20.79 | - | - |
| G23S | 0.66 | 16.00 | - | - |
| L38K | 0.55 | ND | - | - |
| T41A | 2.02 | 22.90 | - | - |
| Q42R | 1.90 | 20.16 | - | - |
| I45F | 0.89 | 26.13 | - | - |
| I45N | - | - | 2.42 | 128.39 |
| I45S | - | - | 2.36 | 74.51 |
| I45T | 3.92 | 37.09 | 24.58 | 232.84 |
| I48T | 0.64 | 8.44 | - | - |
| I56T | 0.70 | 8.64 | - | - |
| M59T | 0.55 | 6.65 | - | - |

| **R1a-A5 output analysis** | | | | |
| --- | --- | --- | --- | --- |
|  | Output I | | Output III | |
| Mutation | Frequency (F, %) | Enrichment factor (E) | Frequency (F, %) | Enrichment factor (E) |
| G13E | - | - | 0.57 | 6.71 |
| W14R | 0.77 | 3.96 | - | - |
| G20E | 1.51 | 48.88 | 0.69 | 22.45 |
| G20R | 0.89 | 32.69 | - | - |
| W21G | 0.77 | 15.63 | 0.63 | 12.92 |
| W21R | 7.85 | 47.99 | 7.99 | 48.82 |
| G23D | 0.96 | 28.13 | - | - |
| G23S | 0.98 | 23.53 | - | - |
| D37G | 0.52 | 5.79 | - | - |
| D37V | 0.50 | 10.11 | - | - |
| I45F | 1.74 | 51.30 | - | - |
| I45S | 0.54 | 16.97 | - | - |
| I45T | 0.85 | 8.02 | - | - |
| I48N | 1.07 | 43.00 | 13.74 | 551.96 |
| I48S | 0.68 | ND | 8.53 | 376.97 |
| I48T | 1.20 | 15.73 | 2.34 | 30.71 |
| T49A | 0.80 | 13.55 | - | - |
| V52E | - | - | 4.69 | 621.29 |
| N53I | 1.40 | 61.79 | - | - |
| N53K | 0.85 | 24.94 | 1.24 | 36.54 |
| N53S | 0.69 | 8.00 | - | - |
| I56N | 0.78 | 39.54 | 1.08 | 55.20 |
| I56S | 0.59 | 46.31 | - | - |
| I56T | 3.41 | 42.29 | 0.83 | 10.26 |

| **R2b-E8 output analysis** | | | | |
| --- | --- | --- | --- | --- |
|  | Output I | | Output III | |
| Mutation | Frequency (F, %) | Enrichment factor (E) | Frequency (F, %) | Enrichment factor (E) |
| W14R | 3.59 | 18.53 | - | - |
| M17T | 1.78 | 21.1 | - | - |
| G20E | 0.71 | 22.84 | - | - |
| W21G | 0.75 | 15.24 | - | - |
| W21R | 5.39 | 32.94 | 4.85 | 29.65 |
| G23S | 0.80 | 23.51 | - | - |
| D37V | 1.08 | 21.64 | - | - |
| T41A | 1.59 | 17.99 | - | - |
| T41S | 1.28 | 32.68 | - | - |
| Q42R | 2.28 | 24.14 | - | - |
| I45A | - | - | 0.73 | ND |
| I45F | 0.87 | 25.72 | 0.80 | 23.69 |
| I45N | - | - | 1.20 | 63.78 |
| I45S | - | - | 2.76 | 87.11 |
| I45T | 3.13 | 29.65 | 22.72 | 215.14 |
| I48N | 0.61 | 24.58 | - | - |
| T49A | 1.31 | 22.3 | - | - |
| N53I | 0.89 | 39.33 | - | - |
| N53K | 0.74 | 21.77 | - | - |
| N53S | 3.37 | 39.22 | - | - |
| N53T | 0.71 | 35.03 | - | - |

| **R1a-G8 output analysis** | | | | |
| --- | --- | --- | --- | --- |
|  | Output I | | Output III | |
| Mutation | Frequency (F, %) | Enrichment factor (E) | Frequency (F, %) | Enrichment factor (E) |
| F9S | 0.43 | 4.11 | - | - |
| W14R | 1.81 | 9.32 | - | - |
| M17K | 5.86 | 85.33 | 22.62 | 329.60 |
| M17R | 1.08 | 23.08 | 7.311 | 156.36 |
| M17T | 1.21 | 14.32 | - | - |
| V18A | 0.50 | 7.79 | - | - |
| V18G | - | - | 0.89 | 34.73 |
| D19E | 0.55 | 13.83 | - | - |
| D19G | 4.10 | 32.91 | - | - |
| D19V | 1.09 | 46.81 | - | - |
| G20E | 1.35 | 43.65 | 1.15 | 37.28 |
| G20R | 0.81 | 29.88 | - | - |
| W21G | 1.26 | 25.72 | 3.79 | 77.38 |
| W21R | 9.56 | 58.40 | 20.52 | 125.40 |
| G23D | 0.93 | 27.32 | - | - |
| G23S | 1.17 | 28.09 | - | - |
| A36V | 0.47 | 14.38 | - | - |
| D37V | 1.09 | 21.85 | - | - |
| I45F | 0.50 | 14.67 | - | - |
| I48T | 1.03 | 13.50 | 0.82 | 10.81 |
| I56T | 0.56 | 6.94 | - | - |

| **FC41 output analysis** | | | | |
| --- | --- | --- | --- | --- |
|  | Output I | | Output II | |
| Mutation | Frequency (F, %) | Enrichment factor (E) | Frequency (F, %) | Enrichment factor (E) |
| G319E (HA1) | 0.54 | 31.05 | 0.39 | 22.22 |
| W14R | 0.87 | 4.50 | 0.78 | 4.00 |
| M17T | 0.52 | 6.18 | 0.53 | 6.29 |
| G20E | 1.91 | 61.73 | 2.64 | 85.51 |
| G20R | 0.69 | 25.34 | 1.01 | 37.11 |
| W21G | 1.369 | 27.93 | 2.61 | 53.26 |
| W21R | 15.07 | 92.07 | 25.51 | 155.85 |
| Y22D | 0.39 | 51.82 | - | - |
| G23D | 1.68 | 49.60 | 2.23 | 65.58 |
| T41A | 1.24 | 14.02 | 0.98 | 11.10 |
| Q42R | 3.40 | 36.05 | 2.95 | 31.31 |
| I45N | 1.07 | 56.73 | 1.64 | 98.59 |
| I45T | 0.81 | 7.69 | 0.67 | 6.33 |
| I48N | - | - | 0.40 | 16.15 |
| I48T | 0.88 | 11.51 | 0.45 | 5.90 |
| I56T | 0.57 | 7.06 | - | - |
| M59T | 0.40 | 5.99 | - | - |

Mutations are listed according to H3 numbering of HA2 domain, except where indicated. Sequencing datasets are available for download through accession number PRJEB15301
