# Supplementary material for: Cross-Neutralising Nanobodies Bind to a Conserved Pocket in the Hemagglutinin Stem Region Identified Using Yeast Display and Deep Mutational Scanning
Source: PLoS One. 2016 Oct 14;11(10):e0164296. doi: 10.1371/journal.pone.0164296 (PMC5065140; doi:10.1371/journal.pone.0164296)
Supplement: S3 Table — (DOCX) [file pone.0164296.s008.docx]

**S3 Table. List of HA2 positions showing residue diversity among group 1 sub-type viral strains (H1, H2, H5 and H9) within residue HA2 Gly^1^-Asn^60^.**

| **Position (HA2)** | **Wild-type residues**  **(H1N1)**  **pdm09** | **Alternative residues (strain, frequency)** | **Residue identified**  **through deep sequencing** | **Position located within or close to sdAb epitope footprint** | **Buried position in HA structure** | **Position in reverse face of HA monomer or at interface of HA trimer** | **Position chosen for mutagenesis** |
| --- | --- | --- | --- | --- | --- | --- | --- |
| 15 | Thr | Gln (H2N2, 100% - H5N1, >99%)  Pro (H9N2, 60%)  Ser (H9N2, 40%) | - | - | - | + | NO |
| 17 | Met | Leu (H9N2, 100%) | + | + | ± | - | YES (M17L) |
| 18 | Val | Ile (H1N1, 4.7%) ^a^ | - | + | - | - | YES (V18I) |
| 19 | Asp | Ala (H9N2, 100%) | - | + | - | - | YES (D19A) |
| 20 | Gly | - ^b^ | + | + | - | - | YES |
| 21 | Trp | - ^b^ | + | + | - | - | YES |
| 24 | Tyr | Phe (H5N1, 5.1% - H9N2, 100%) | - | - | ± | - | YES (Y24F) |
| 25 | His | Gln (H9N2, 100%) | - | - | - | + | NO |
| 27 | Gln | Ser (H5N1, >99%  H2N2, 100% - H9N2, 100%) | - | - | - | + | NO |
| 29 | Glu | Asp (H2N2, 98.6% - H9N2, 100%) | - | - | - | + | NO |
| 32 | Ser | Val (H9N2, 100%) | - | - | - | + | NO |
| 34 | Tyr | Met (H9N2, 100%) | - | + | - | - | YES (Y34M) |
| 38 | Leu | Gln (H1N1, 24.4%)  Lys (H5N1, 96.9% - H2N2, 100%)  Arg (H9N2, 100%) | - | + | - | - | YES (L38Q,K,R) |
| 39 | Lys | Glu (H5N1, >99% - H2N2, 100%)  Asp (H9N2, 100%) | - | - | - | + | NO |
| 43 | Asn | Lys (H5N1, 93.13% - H2N2, 95.8% - H9N2, 100%)  Arg (H5N1, 6.44% - H2N2, 4.2%) | - | - | - | + | NO |
| 45 | Iso | Phe (H2N2, 100%)  Val (H9N2, 20%) | + | + | - | - | YES (I45F,V) |
| 46 | Asp | Asn (H1N1, 23.3%) | - | + | - | - | YES (D46N) |
| 47 | Glu | Lys (H1N1, 34.8% - H9N2, 100%) ^c^  Gly (H1N1, 24.1% - H5N1, >99% - H2N2, 83.3%)  Arg (H2N2, 13.9%) | - | - | - | + | YES (E47K) |
| 48 | Ile | Val (H5N1, >99%) | + | + | - | - | YES (I48V) |
| 50 | Asn | Ser (H9N2, 100%) | - | + | - | - | YES (N50S) |
| 52 | Val | - ^b^ | + | + | - | - | YES |
| 53 | Asn | - ^b^ | + | + | - | - | YES |
| 54 | Ser | Asn (H9N2, 100%) | - | - | - | + | NO |
| 55 | Val | Ile (H5N1, >99%) | - | + | + | - | YES (V55I) |
| 56 | Ile | Val (H9N2, 100%) | - | + | - | - | YES (I56V) |
| 57 | Glu | Asp (H5, 94.3%)  Asn (H5, 5.2%)  Lys (H9N2, 100%) | - | + | - | - | YES (E57D,N) |

^a^ Ile mutation frequency below the threshold, but positions chosen because it falls within the epitope footprint

^b^ Residues absolutely conserved in H1,H2,H5, H9 subtypes and identified by deep sequencing as a key antibody epitope residue

^c^ E47K is a fast growing mutation in post-2009 pandemic H1N1 strains [1].

^Reference List^

^1. Maurer-Stroh S, Lee RT, Eisenhaber F, Cui L, Phuah SP, Lin RT (2010) A new common mutation in the hemagglutinin of the 2009 (H1N1) influenza A virus. PLoS Curr 2: RRN1162. k/-/-/3rs9roocl5v8f/1 [pii].^
