# Supplementary material for: Cross-Neutralising Nanobodies Bind to a Conserved Pocket in the Hemagglutinin Stem Region Identified Using Yeast Display and Deep Mutational Scanning
Source: PLoS One. 2016 Oct 14;11(10):e0164296. doi: 10.1371/journal.pone.0164296 (PMC5065140; doi:10.1371/journal.pone.0164296)
Supplement: S4 Table — (DOCX) [file pone.0164296.s009.docx]

**Table S4. List of mutation in the unselected library identified by deep sequencing (HA1 Gly^303^-HA2 Asn^71^)**

| HA domain | HA1 | | | | | | | | | | | | | | | | | | | | | | | | | | | HA2 | | | | |
| --- | --- | --- | --- | --- | --- | --- | --- | --- | --- | --- | --- | --- | --- | --- | --- | --- | --- | --- | --- | --- | --- | --- | --- | --- | --- | --- | --- | --- | --- | --- | --- | --- |
| Position | 303 | | | | | | | 310 | | | | | | | | | | 320 | | | | | | | | | | 1 | | | | |
| Wild-type residue | G | K | C | P | K | Y | V | K | S | T | K | L | R | L | A | T | G | L | R | N | I | P | S | I | Q | S | R | G | L | F | G | A |
| Mutations sampled | A | E | F | A | N | C | A | E | C | A | D | F | G | M | D | A | A | C | G | D | F | A | A | F | H | A | G | A | I | C | A | D |
|  | E | N | G | L | Q | D | E | I | G | I | E | M | I | P | E | I | E | F | K | H | L | L | C | L | K | F | I | C | P | I | E | G |
|  | R | Q | R | Q | R | F | G | N | I | K | I | S | K | Q | G | K | R | M | M | I | M | Q | F | M | L | P | K | D | Q | L | R | P |
|  | V | R | S | R | T | H | I | Q | N | P | N | V | S | R | P | P | V | S | S | K | N | R | P | N | P | T | S | R | R | S | S | S |
|  |  | T | W | S | E | S | L | R | R | R | Q | W | T |  | S | R |  | V | T | S | S | S | T | S | R | Y | T | S | V | T | V | T |
|  |  |  | Y | T | I |  |  | T | T | S | R |  |  |  | T | S |  | W | V | T | T | T | Y | T |  |  |  | V |  | V | W | V |
|  |  |  |  |  |  |  |  |  |  |  | T |  |  |  | V |  |  |  | W | Y | V |  |  | V |  |  |  |  |  | W |  | Y |
|  |  |  |  |  |  |  |  |  |  |  |  |  |  |  |  |  |  |  |  |  |  |  |  |  |  |  |  |  |  | Y |  |  |

| HA domain | HA2 | | | | | | | | | | | | | | | | | | | | | | | | | | | | | | | |
| --- | --- | --- | --- | --- | --- | --- | --- | --- | --- | --- | --- | --- | --- | --- | --- | --- | --- | --- | --- | --- | --- | --- | --- | --- | --- | --- | --- | --- | --- | --- | --- | --- |
| Position | 6 | | | | 10 | | | | | | | | | | 20 | | | | | | | | | | 30 | | | | | | | |
| Wild-type residue | I | A | G | F | I | E | G | G | W | T | G | M | V | D | G | W | Y | G | Y | H | H | Q | N | E | Q | G | S | G | Y | A | A | D |
| Mutations sampled | F | D | A | C | F | A | A | A | C | A | A | E | A | A | A | C | C | A | C | D | D | E | D | A | E | A | A | E | C | E | D | A |
|  | G | G | C | D | L | D | E | E | G | E | E | G | E | E | E | G | D | C | D | L | L | H | H | D | H | E | L | R | D | G | G | E |
|  | L | P | D | I | M | G | R | R | L | I | R | I | G | G | R | L | F | D | F | N | N | K | I | G | K | R | P | V | F | P | P | G |
|  | M | S | S | L | N | K | V | V | R | K | V | K | I | N | V | R | G | R | H | P | P | L | K | K | L | V | T |  | H | S | S | H |
|  | N | T | V | P | S | V | W | W | S | P | W | L | K | V |  | S | H | S | N | Q | Q | P | S | Q | P | W |  |  | N | T | T | K |
|  | S | V |  | S | T |  |  |  | V | R |  | R | L | Y |  |  | N | V | S | R | R | R | T | V | R |  |  |  | S | V | V | N |
|  | T |  |  | V | V |  |  |  |  | S |  | T |  |  |  |  | S |  |  | Y | Y |  | Y |  | S |  |  |  |  |  |  | V |
|  | V |  |  | Y |  |  |  |  |  |  |  | V |  |  |  |  |  |  |  |  |  |  |  |  |  |  |  |  |  |  |  | Y |

| HA domain | HA2 | | | | | | | | | | | | | | | | | | | | | | | | | | | | | | | | | |
| --- | --- | --- | --- | --- | --- | --- | --- | --- | --- | --- | --- | --- | --- | --- | --- | --- | --- | --- | --- | --- | --- | --- | --- | --- | --- | --- | --- | --- | --- | --- | --- | --- | --- | --- |
| Position | 38 | | 40 | | | | | | | | | | 50 | | | | | | | | | | 60 | | | | | | | | | | 70 | |
| Wild-type residue | L | K | S | T | Q | N | A | I | D | E | I | T | N | K | V | N | S | V | I | E | K | M | N | T | Q | F | T | A | V | G | K | E | F | N |
| Mutations sampled | M | E | C | A | E | D | D | F | A | A | F | A | D | E | A | D | A | A | E | A | E | I | D | A | E | C | A | E | A | A | E | A | C | D |
|  | P | M | G | E | H | H | G | L | E | D | L | I | H | G | E | F | C | D | G | D | M | K | G | I | H | G | I | P | E | C | I | D | G | I |
|  | Q | N | I | I | K | I | P | M | G | G | M | N | I | I | G | H | F | G | L | G | N | L | H | K | K | I | K | S | G | D | N | G | I | K |
|  | R | Q | N | K | L | K | S | N | N | K | N | P | K | N | I | I | P | I | M | K | Q | R | I | P | L | L | P | T | I | R | Q | K | L | S |
|  | S | R | R | P | P | S | T | P | V | Q | S | S | S | R |  | K | T |  | N | Q | R | T | K | R | P | S | R | V | L | S | R | Q | S | T |
|  | V | T |  | S | R | T | V | S | Y | V | T |  | T | Q |  | S | Y |  | S | V | T | V | S | S | R | V | S |  |  | V | T | V | V | Y |
|  |  |  |  |  |  | Y |  | T |  |  | V |  | Y | T |  | T |  |  | T |  |  |  | T |  |  | Y |  |  |  | W |  |  | Y |  |
|  |  |  |  |  |  |  |  | V |  |  |  |  |  |  |  | Y |  |  | V |  |  |  | Y |  |  |  |  |  |  |  |  |  |  |  |

Sequencing datasets are available for download through accession number PRJEB15301
